# Disease Outbreak, Health Scare, and Distance Decay: Evidence from HPAI Shocks in Chinese Meat Sector

**DOI:** 10.3390/ijerph17218009

**Published:** 2020-10-30

**Authors:** Lan Yi, Congcong Duan, Jianping Tao, Yong Huang, Meihua Xing, Zhongkun Zhu, Caifeng Tan, Xinglin Chen

**Affiliations:** 1 Institute of Agricultural Economics & Technology, Hubei Academy of Agricultural Sciences, Wuhan 430064, China; yilan@webmail.hzau.edu.cn (L.Y.); wihzauxmh@126.com (M.X.); 2015202050114@whu.edu.cn (X.C.); 2 Sub-Center for Agricultural Economics & Technology, Hubei Center for Agricultural Science & Technology Innovation, Wuhan 430064, China; 3 Hubei Academy of Rural Revitalization, Wuhan 430064, China; 4 College of Economics & Management, Huazhong Agricultural University, Wuhan 430070, China; huangyong@mail.hzau.edu.cn (Y.H.); tancaicai@webmail.hzau.edu.cn (C.T.); 5 Hubei Rural Development Research Center, Wuhan 430070, China; 6 National School of Development, Peking University, Beijing 100871, China; zzk@mail.hzau.edu.cn; 7 Department of Agricultural and Resource Economics, North Carolina State University, Raleigh, NC 27695, USA

**Keywords:** zoonotic disease shock (ZDS), zoonotic disease outbreak (ZDO), public health scare (PHS), meat price risk (MPR), distance-decaying spillover, spatial attenuation boundaries, highly pathogenic avian influenza (HPAI), spatial spillover measures, distance-varying spatial weighting matrix

## Abstract

*Background:* During zoonotic disease shocks (ZDSs), zoonotic disease outbreaks (ZDOs) can induce public health scares (PHSs), causing meat price risks (MPRs). Nevertheless, spatial spillovers of zoonotic disease shocks in meat markets remain unclear. We explore how zoonotic disease outbreaks and public health scares locally and spatially spill over to meat price risks, and whether spatial spillovers of public health scares decay with distance. *Methods:* (i) We construct a long panel covering 30 provinces and 121 months, using highly pathogenic avian influenza (HPAI) epidemics as exogenous shocks in Chinese meat sector. (ii) We decompose zoonotic disease shocks into zoonotic disease outbreaks (objective incident) and public health scares (subjective information) and examine their spillovers to meat price risks. (iii) We identify distance-decaying spatial spillovers of public health scares, by running our dynamic SAR models 147 times, from 80 km to 3000 km with 20 km as incremental value, in a setting with risk-level heterogeneity. *Results:* (i) Zoonotic disease outbreaks themselves only cause local and neighboring meat price risks for high-risk meat, not for low-risk or substitute meat. (ii) Public health scares exacerbate local and neighboring meat price risks for high-risk and low-risk meat, and local meat price risks for substitute meat. (iii) Spatial spillovers of public health scares are distance-decaying and U-shaped, with four spatial attenuation boundaries, and distance turning point is shorter for high-risk meat (500 km) than for low-risk meat (800 km). *Conclusions:* We complement the literature by arguing that health scares induced by disease outbreaks negatively spill over to meat prices, with U-shaped distance-decaying spatial effects. This suggests low interregional spatial market integration in meat products, due to distance decay of nonstandardized information and local government control effects, across provincial boundaries. To the best of our knowledge, we are the first to document nonmonotonic distance decay of health scare effects on food prices, previously not found by the literature.

## 1. Introduction

Zoonotic disease poses a critical threat to sustainable public health [1]. Since the 20th century, epidemic animal diseases occur all around the world, including zoonotic diseases, which can seriously threaten human health and animal husbandry [2]. In fact, zoonotic disease control and prevention are extremely arduous due to various sources of infection and complex channels of transmission [3]. Even worse, zoonotic diseases directly harm individual health and safety, and thus severely challenge the long-term sustainability of public health. To illustrate, some animal-only viral diseases (such as bovine spongiform encephalitis (BSE), foot-and-mouth disease (FMD), and highly pathogenic avian influenza (HPAI)) gradually mutate into human infectious diseases (i.e., zoonotic diseases), endangering human lives [4]. For instance, avian influenza viruses are highly species-specific, but have, in recent years, crossed the species barrier to infect humans (e.g., H5N1 and H7N9), jeopardizing human well-being and food safety [5]. According to official statistics, human infections with H5N1 occur across 16 countries and regions worldwide, with 856 global confirmed human infection cases reported by 16 January 2017; while human infections with H7N9 occur mostly in China, with 1115 global confirmed human infection cases reported by 8 January 2017. As covered by European Food Safety Authority (EFSA), from 16 November 2019 to 15 February 2020, 36 HPAI A(H5N8) outbreaks were reported in Europe in poultry (*n* = 34), wild birds (*n* = 2), and captive birds (*n* = 1), in Germany, Romania, Poland, Hungary, Slovakia, Czechia, and Ukraine; one HPAI outbreak caused by A(H5N2) and A(H5N8) was reported in poultry in Bulgaria; two A(H5) outbreaks were reported in poultry in Denmark and the United Kingdom; an increasing number of HPAI A(H5N1), A(H5N2), A(H5N5), and A(H5N6) outbreaks in poultry in Asia were reported compared with the previous reporting period; and two human infection cases caused by A(H9N2) were reported during this period [6].

Zoonotic disease outbreaks (ZDOs) can lead to public health scares (PHSs) [7]. With the rapid development of information and communication technology (ICT), information on disease epidemics spreads much faster in the Internet era than before. Further, the impacts of zoonotic disease outbreaks could be aggravated by substantial online news and social media coverage, online consumers’ special attention to epidemics, and individuals’ active online searches for information about disease outbreaks [8]. Actually, it is often difficult for individuals to know the truth about food safety incidents due to information asymmetry in food markets; accordingly, individuals tend to rely more on low-cost and convenient information channels such as public opinion, and employ media coverage as main news and information sources for obtaining information on the incidents and analyzing its effects [9]. Moreover, online news and social media can transmit information about food safety risks, and draw consumers’ attention and awareness to zoonotic disease outbreaks; this may significantly raise individuals’ risk perceptions and even lead to mass consumers’ herding behavior, resulting in widespread public health scares [10].

Zoonotic disease shocks (ZDSs) may cause meat price risks (MPRs) [11]. Indeed, prices of livestock and poultry products would respond quickly to abrupt zoonotic disease shocks, and rise and fall suddenly and sharply quite often during epidemics [12]. For example, Chinese broiler chick prices fell from 2.55 RMB per kilogram to 1.42 RMB per kilogram, in response to the H5N1 shock in China’s poultry sector in May 2005; similarly, Korean dressed broiler prices collapsed immediately following the avian influenza shock in South Korea’s consumer markets in November 2016, and subsequently soared to a 30-year high in March 2017. As described, zoonotic disease shocks can give rise to extreme volatility in related meat prices, likely because the adverse impacts of zoonotic disease outbreaks might be even aggravated by public health scares, which may substantially exacerbate meat price risks in consumer markets [13]. In summary, zoonotic disease shocks and their consequences are not only increasingly recognized as a serious, worldwide public health concern, but also a major threat to meat sectors.

Risk levels can be heterogeneous across meat products, both for the meat industry and for meat consumption [14]. For instance, at different parts of poultry meat supply chain, diverse poultry products could have heterogeneous risk levels [15]. Live broiler, a midstream product in poultry meat supply chain, may be relatively high-risk to consume, since avian influenza viruses, such as H5 subtype of HPAI and H7N9, are substantially more likely to infect human from live broiler, causing foodborne diseases (FBDs) [16]. Thus, live broiler in midstream poultry meat supply chain can be a high-risk meat product [17]. On the other hand, dressed broiler in downstream poultry meat supply chain may be relatively low-risk to consume, because dressed broiler production can be large-scale and standardized, and quality and safety control of dressed broiler is relatively strict [18]. Hence, dressed broiler in downstream poultry meat supply chain can be a low-risk meat product. It is worth noting that although the consumption of live broiler could be more risky than dressed broiler (especially during HPAI epidemics), live broiler consumption is still prevalent in some developing countries like China, where live broiler markets are not completely closed. Meanwhile, in most cases, pork might be substitute meat for poultry products in China, in that a decrease in poultry consumption usually leads to an increase in pork consumption.

There are a large number of published studies that describe the economic impacts of zoonotic disease shocks in food markets [19,20,21,22,23]. However, in previous literature, (i) evidence for the individual effects of zoonotic disease outbreaks and public health scares in meat markets has been mixed; (ii) it is still not known whether the relevant causal effects are heterogeneous among high-risk, low-risk, and substitute meat products, and between high-risk and low-risk disease outbreaks; (iii) the spatial spillovers of zoonotic disease outbreaks and public health scares to meat prices remain unclear; and (iv) very little is known about the distance decay of public health scare effects on meat price risks.

The purpose of this study is to fill these knowledge gaps by determining whether (i) zoonotic disease outbreaks have negative local and spatial spillovers to meat price risks; (ii) public health scares have negative local and spatial spillovers to meat price risks; (iii) spatial spillovers of public health scares to meat price risks are distance-decaying with spatial attenuation boundaries; and (iv) the spillovers are heterogeneous across high-risk, low-risk, and substitute meat.

This article investigates three research questions.

**Q1** **(price risk spillover):** *How do zoonotic disease outbreaks and public health scares locally and spatially spill over to meat price risks, respectively?*

**Q2** **(distance-decaying spillover):** *Do spatial spillovers of public health scares to meat price risks decay with distance?*

**Q3** **(risk spillover heterogeneity):** *Are the spillovers heterogeneous across meat products?*

In this paper, we propose and implement a novel approach to detect potential nonmonotonic distance decay of health scare effects on food prices. Using HPAI epidemics as exogenous shocks in Chinese meat sector, with a long spatial panel for 30 provinces and 121 months, we decompose zoonotic disease shocks into zoonotic disease outbreaks (objective incident component) and public health scares (subjective information component), examine their local and spatial spillovers to meat price risks, and further identify distance-decaying spatial spillovers of public health scares to meat price risks, by running our dynamic SAR models 147 times, from 80 km to 3000 km with 20 km as incremental value, in a setting with heterogeneous risk-level meat products and disease outbreaks.

We document five new stylized facts. First, zoonotic disease outbreaks themselves only cause local and neighboring meat price risks for high-risk meat, not for low-risk or substitute meat. (i) For high-risk meat, both high-risk and low-risk zoonotic disease outbreaks have negative local and spatial spillovers to meat price risks; and (ii) for low-risk and substitute meat, neither high-risk nor low-risk zoonotic disease outbreaks have significant local or spatial spillovers to meat price risks.

Second, public health scares exacerbate local and neighboring meat price risks for high-risk and low-risk meat, and local meat price risks for substitute meat. (i) For high-risk and low-risk meat, public health scares over zoonotic disease have negative local and spatial spillovers to meat price risks; and (ii) for substitute meat, public health scares over zoonotic disease only have negative local spillovers to meat price risks, while spatial spillovers of public health scares to meat price risks are statistically insignificant.

Third, spatial spillovers of public health scares are distance-decaying and U-shaped. The negative short-run spatial spillovers of public health scares over zoonotic disease to meat price risks are distance-decaying, and the short-run effects of distance on spatial spillovers of public health scares to meat price risks are U-shaped, where there exist distance-decaying spatial attenuation boundaries: The distance turning point divides the U-shape into the strengthening region and the weakening region, which are further subdivided into the enhancing regime, the recovering regime, the half-decaying regime, and the slow-decaying regime.

Fourth, distance effects on spatial spillovers of public health scares are heterogeneous across meat products. (i) The distance turning point is lower for high-risk meat (500 km) than it is for low–risk meat (800 km); and (ii) as spatial spillovers of public health scares to substitute meat price risks are statistically insignificant, there exist no distance-decaying spatial attenuation boundaries for substitute meat.

Fifth, public health scares maintain long-run impacts on local meat price risks for low-risk meat. In the long run, only local spillovers of public health scares to low-risk meat price risks are significantly negative, while others are statistically insignificant.

Our study is important because we contribute to the literature on the economic impacts of disease shocks in several ways. First, disease shock decomposition. In contrast to early work where zoonotic disease shocks are considered in their entireties, we decompose zoonotic disease shocks into zoonotic disease outbreaks (objective incident component) and public health scares (subjective information component), and gauge respectively the individual effects of zoonotic disease outbreaks and public health scares on meat price risks, instead of the aggregate effects of zoonotic disease shocks, so as to better understand how meat markets respond to incident and information shocks during epidemics.

Second, risk-level heterogeneity. Different from existing work where meat products and disease outbreaks are in a setting with homogeneous risk levels, we use a setting with risk-level heterogeneity, and distinguish among high-risk, low-risk, and substitute meat products (as measured by live broiler, dressed broiler, and pork, respectively), and between high-risk and low-risk disease outbreaks (as measured by human and poultry infections with HPAI outbreaks, respectively), so as to better measure the causal impacts of zoonotic disease outbreaks and public health scares on meat price risks across heterogeneous risk levels in disease outbreaks and meat products, which complements existing methods.

Third, spatial spillover effects. Compared with existing literature where meat price volatility and transmission based on time series regressions are of great interest, we restrict attention to local and spatial spillovers of zoonotic disease outbreaks and public health scares to meat price risks using dynamic spatial models, which remains largely unexplored.

Fourth, spatial distance decay. Contrasting with previous research where distance effects on food prices are estimated by means of simple regressions, without accounting for distance-decaying spatial effects, we create distance-varying spatial weighting matrix and spatial spillover measures weighted by distance, and run regressions of high- and low-risk meat price risks 147 times, respectively, from 80 km to 3000 km with 20 km as incremental value, implementing dynamic SAR, so as to identify nonmonotonic distance decay of spatial spillovers of health scares to meat prices, with spatial attenuation boundaries, previously undocumented in the literature to date.

Throughout this paper, (i) “zoonotic disease shocks (ZDSs)” refer to the issues of zoonotic diseases as whole, which can be decomposed into zoonotic disease outbreaks (ZDOs, objective incident component) and public health scares (PHSs, subjective information component) over zoonotic disease; (ii) “zoonotic disease outbreaks (ZDOs)” refer to the objective incident component of zoonotic disease shocks, and are divided into high–risk zoonotic disease outbreaks (as measured by human infections with HPAI outbreak dummy) and low–risk zoonotic disease outbreaks (as measured by poultry infections with HPAI outbreak dummy); (iii) “public health scares (PHSs)” refer to the subjective information component of zoonotic disease shocks, and are measured by log Baidu search volumes on HPAI; (iv) “meat price risks (MPRs)” mean that zoonotic disease shocks spill over to the livestock and poultry sectors and lead to price risks in meat markets, and are divided into high-risk meat price risks (as measured by log live broiler prices), low-risk meat price risks (as measured by log dressed broiler prices), and substitute meat price risks (as measured by log pork prices); (v) “distance-decaying spillovers” mean that the spatial spillovers of public health scares over zoonotic disease to meat price risks decay with geographic distance; (vi) “spatial attenuation boundaries” mean that the distance-decaying spatial spillovers of public health scares over zoonotic disease to meat price risks are U-shaped, which are subdivided into four regimes (enhancing regime, recovering regime, half-decaying regime, and slow-decaying regime); and (vii) “spatial spillover measures” refer to short-run spatial spillover effects of public health scares on meat price risks, at different distance thresholds.

**Figure 1:**
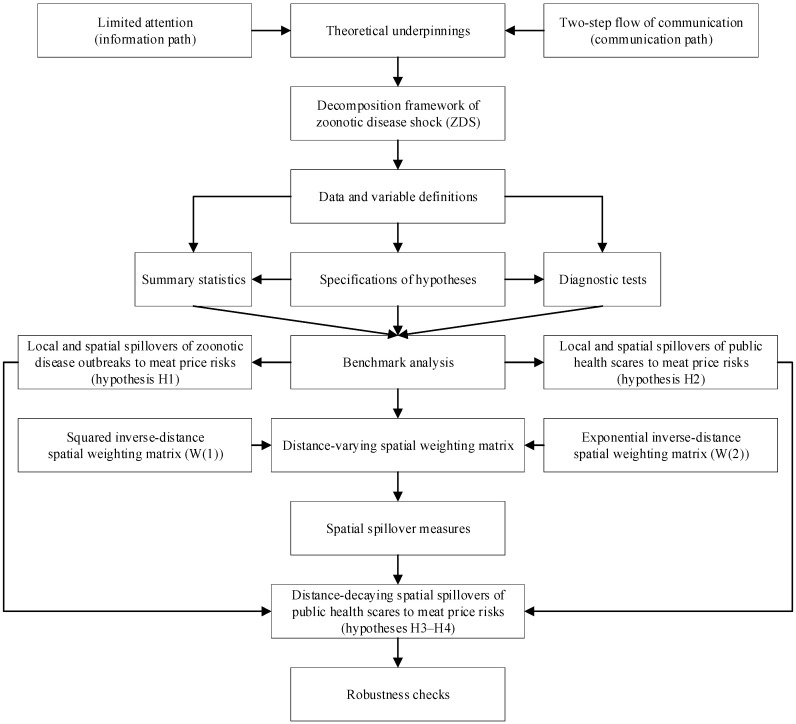
Flowchart of empirical research. The figure shows our research procedures in this article. Source: Originally developed by the authors.

The rest of the paper is organized as follows. Section 2 reviews the related literature, provides theoretical underpinnings by developing a decomposition framework of zoonotic disease shocks, and formulates hypotheses. Section 3 presents the research design, the data collected, variable definitions, and empirical specifications. Section 4 presents our diagnostic tests, main empirical results, and robustness checks. Section 5 discusses the relation with the existing literature and interpretations of results. Section 6 concludes with a discussion of policy implications and future research avenues. We employ a flowchart of empirical research to illustrate our research procedures (Figure 1).

## 2. Theory

### 2.1. Theoretical Underpinnings

This article connects to three strands of literature. First, there is a large body of literature on spillovers of zoonotic disease outbreaks to meat price risks. Our hypothesis that zoonotic disease outbreaks have negative spillover to meat price risks is associated with Nina et al. [19] claiming that brucellosis in cattle significantly lowers milk price, employing semi-structured interviews with serological data in south western Uganda; is consistent with that of Govindaraj et al. [11] arguing that avian influenza outbreaks may cause a sharp decline in duck meat and other poultry product prices, a transient increase in substitute product (beef and fish products) prices, and an eight-week lag in price recovery, based on data from pre-tested schedules and developmental departments in Kerala, India; confirms McLachlan and Yestrau [24] suggesting that bovine spongiform encephalopathy (BSE) can lead to low cattle commodity prices, using surveys across western Canada; and accords with Weber [20] showing that paratuberculosis in dairy herds can result in a reduction of milk price, adopting the risk management method.

The second strand of literature is on spillovers of public health scares to meat price risks. Our hypothesis that public health scares have negative spillovers to meat price risks, reflects those of Hassouneh, Serra, and Gil [12] finding that BSE food scares have a negative influence on beef producer prices and no influence on beef consumer prices, utilizing a regime-switching vector error correction model with a BSE food scare index in the Spanish bovine sector; corroborates Serra [13] maintaining that the impact of BSE food scares on food price volatility leads to two price behavior regimes, one where food scare news grow with negative correlation between producer and consumer price volatility, and another where food scare news decline with positive correlation between producer and consumer price volatility, relying upon a smooth transition conditional correlation (STCC) GARCH model with the case of the BSE in Spain; broadly supports the work of Hassouneh et al. [21] concluding that avian influenza (AI) food scare crises have a heterogeneous impact on vertical poultry price transmission, where wholesaler margins fall while retailers’ marketing margins rise, utilizing a bivariate smooth transition vector error correction model (STVECM) with an AI food scare information index in the Egyptian poultry sector; is in accordance with Lloyd et al. [22] pointing out that BSE food scares have a more-than-twofold impact on farm beef prices than that on retail beef prices due to retail market power, proposing impulse-response analysis with multivariate models during the UKBSE crisis; is consistent with Lloyd et al. [23] maintaining that BSE food scares have a negative impact on producer, wholesale, and retail beef prices, which drop by 3.0, 2.25, and 1.70 pence/kg, respectively, owing to market power, employing a co-integrating framework with the ‘food publicity’ index in the UK beef market; supports evidence from Capps, Colin-Castillo, and Hernandez [25] holding the view that food scares caused by Food Safety Inspection Service (FSIS) recalls have no impact on beef and pork marketing margins, but with small cross-effects from pork to beef, while food scares caused by BSE outbreaks have a significant impact on wholesale-to-retail beef marketing margins and wholesale-to-retail beef and pork price transmission, using monthly national data spanning 1986–2008 in the U.S. red meat industry.

There is also a connection to the literature on distance-decaying spillovers of public health scares to meat price risks. Our hypothesis that meat price risks are distance-decaying, is consistent with Cudjoe, Breisinger, and Diao [26] maintaining that distance between producer and consumer markets determines food price transmission, employing a threshold cointegration model in Ghana; consistent with Iregui and Otero [27] arguing that distance, namely transportation costs, negatively affects the speed of food price adjustment to shocks in other areas, based on generalized impulse response analysis with a highly disaggregated dataset for Colombia; consistent with Singh-Peterson et al. [28] concluding that distance from food distribution center positively affects food price, using a spatial analysis of a healthy food basket survey undertaken across Queensland, Australia; consistent with Palermo et al. [29] pointing out that distance from state capital city center is positively related to food price, adopting standard multiple regressions and multi-collinearity tests in Victoria; consistent with Yan, Terheggen, and Mithofer [30] asserting that distance between farmers’ locations and nearest village market negatively affect village-level food price, utilizing a multiple regression analysis for small-scale farmers in Southwest China; consistent with Le Cotty, d’Hotel, and Ndiaye [31] claiming that market remoteness measured by distance, i.e., transport costs, has a positive effect on food price volatility, relying upon an autoregressive conditional heteroskedasticity model in Africa; and consistent with Iregui and Otero [32] suggesting that for traded food products, distance is negatively associated with the speed of food price adjustment to the long-run equilibrium, utilizing a pairwise approach to testing for spatial market integration in Colombia.

Previous studies have generally relied on the efficient market hypothesis (EMH) [33], which assumes that consumers can rationally react to market information. These studies typically consider zoonotic disease shocks simply as whole, without differentiating their potential components, and concentrate on quantifying the impacts of zoonotic diseases on meat price fluctuations and transmission, thus actually analyzing the total effects of zoonotic disease shocks on meat price risks.

**Figure 2:**
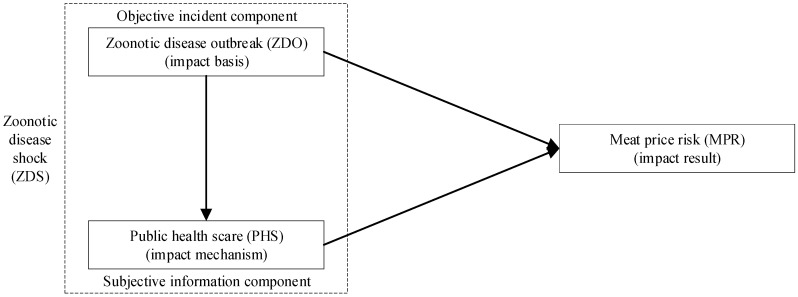
Decomposition framework of zoonotic disease shock (ZDS). The figure shows that (i) zoonotic disease shocks (ZDSs) can be decomposed into zoonotic disease outbreaks (ZDOs, objective incident component) and public health scares (PHSs, subjective information component); and (ii) zoonotic disease outbreaks (ZDOs, impact basis) can either directly affect meat price risks (MPRs, impact result), or indirectly affect meat price risks (MPRs, impact result) through public health scares (PHSs, impact mechanism). Source: Originally developed by the authors.

We introduce theories of limited attention [34] and two-step flow of communication [35], and decompose zoonotic disease shocks into zoonotic disease outbreaks (objective incident component) and public health scares (subjective information component), so that we can clearly distinguish and isolate the spillovers of the components to meat price risks (Figure 2).

Following our prior work [36,37], we incorporate paths of information and communication into the classical theoretical framework of food price fluctuations and transmission. (i) In the age of big data, when zoonotic disease outbreaks occur, Internet media, who serve as online opinion leaders, disseminate zoonotic disease information during second-step flow. (ii) The upsurge of online media coverage arouses bounded rational consumers’ public health scares over zoonotic disease. (iii) Agglomeration of consumers’ behavioral biases can lead to herding behavior, which in turn may cause meat market overreaction. (iv) Consumers’ behavioral biases and market overreaction can thus amplify the local and spatial spillovers of zoonotic disease outbreaks to meat price risks.

### 2.2. Hypotheses

Zoonotic disease outbreaks, the exogenous incidents, exert influences on meat sectors, both on the supply side and demand side. (i) On the supply side, zoonotic disease outbreaks can kill and lead to the forced culling of dozens of millions of livestock, dramatically decreasing livestock inventory; moreover, livestock breeding and trading may be temporarily prohibited shortly after the disease epidemic, restricting the circulation of livestock products, which limits market integration and lowers the effective supply of livestock meat. (ii) On the demand side, zoonotic disease outbreaks are sudden incidents, and information on zoonotic disease is released by the authorities or official media; flooded by an enormous amount of information, the public may underreact to the source information on zoonotic disease, due to bounded rationality; therefore, risk perceptions of zoonotic disease may lower consumers’ willingness to pay (WTP), while it has a very limited impact on consumer markets.

For high-risk meat products (such as live broiler), consumers might dramatically decrease consumption, for fear of getting infected with zoonotic diseases; and live broiler demand decrease can significantly surpass supply decrease, thus having negative impact on live broiler prices. Moreover, the price risk of live broiler might spill over to neighboring poultry markets via interregional horizontal price transmission mechanism, owing to meat spatial market integration, that is, the law of one price (LOP) across meat markets. Meanwhile, for low-risk meat products (such as dressed broiler), dressed broiler supply and demand decreases could counteract each other; hence, the price risk of dressed broiler might be insignificant.

Taken together, the effect of supply reduction may partially offset the effect of demand reduction caused by limited attention of bounded rational consumers. Therefore, zoonotic disease outbreaks could pose heterogeneous impacts on meat price risks, i.e., changes in equilibrium prices might be heterogeneous across high-risk, low-risk, and substitute meat. Furthermore, considering that zoonotic disease outbreaks may not only spill over to local meat price risks, but also to neighboring meat price risks owing to the spatial spillover effects, we propose Hypothesis H1.

**Hypothesis** **1** **(H1):** *Zoonotic disease outbreaks have negative local and spatial spillovers to meat price risks, which are heterogeneous across high-risk, low-risk, and substitute meat.*

The classical theory of complete markets assumes complete information and full rationality, and price is an accurate reflection of product information; since products accurately reflect their true value and complete information, and media reports do not affect changes in market prices, the price effects of media should be insignificant. However, the existence of transaction costs and consumers’ bounded rationality may call into question whether the efficient market hypothesis from the classical theory of complete markets holds, and the price effects of media information and communication under the hypothesis of bounded rationality has attracted much academic interest.

Previous studies maintain that zoonotic disease outbreaks exert impacts on livestock product markets, and the negative media coverage on zoonotic disease can lead to price fluctuations. In the age of big data, online media in various forms can rapidly report and transmit incidents, improving the speed and coverage of information flows; the diversification of online media entities and communication forms accelerates the spread of information, leading to more significant effects of online media on consumer markets; the effects of online media often express themselves as the rapid propagation of information, resulting in attention clustering. When zoonotic disease outbreaks occur, online news media and social media can identify the hot incident, pick out the source information on zoonotic disease from massive information, and then retransmit the source information, resulting in public health scares over zoonotic disease; online attention to negative information may influence consumers’ future expectations directly through consumer markets. Under the influence of public health scares over zoonotic disease, consumers’ attention, confidence, and sentiment are deeply affected, thus enhancing risk perceptions of zoonotic disease; bounded rationality may lead to consumers’ behavioral bias, that is, overreaction to consumption risk of livestock products; therefore, mass consumers’ clustered behavioral bias may cause mass market panics, which could wield a great influence over livestock market demand. Although both supply and demand of livestock products decline, consumers may dramatically decrease livestock demand due to public health scares over zoonotic disease, therefore the decrease in demand could exceed the decrease in supply of livestock products, leading to the livestock price drop, which has a considerable impact on livestock price fluctuations, resulting in livestock price risk. In a real market setting, consumers may have different risk estimates during first-step flow and second-step flow of zoonotic disease information owing to bounded rationality and limited information, and overreact to zoonotic disease shocks, resulting in price risk in livestock markets.

According to the existing literature, public health scares over zoonotic disease could have cross-regional impacts on neighboring livestock markets, leading to consumers’ overreaction, with a significant reduction in consumption, which results in a negative spatial spillover to neighboring livestock prices. Because online information transmits fast across individuals and areas by online news and social media, general public’s health scares over zoonotic disease might spread to neighboring regions and lead to meat price risks in nearby markets. In addition, local prices of substitute meat could also be influenced by public health scares, due to industrial vertical meat price transmission mechanism. Thus, we allow for the spatial spillover effects of public health scares to meat price risks and propose Hypothesis H2.

**Hypothesis** **2** **(H2):** *Public health scares have negative local and spatial spillovers to meat price risks, which are heterogeneous across high-risk, low-risk, and substitute meat.*

Recent evidence suggests that in the fields of economics and market behavior, the spatial spillover effects among economic variables may have certain regional boundaries; geographic distance is a key determinant of spatial spillover effect, where the spatial spillover effect attenuates with the increase in the geographic distance, that is, spatial spillover effect is negatively associated with geographic distance [38,39,40,41,42].

Based on Hypothesis H2, public health scares over zoonotic disease have spatial spillovers to meat price risks, and here we further consider the geographic boundaries of spatial spillovers of public health scares to meat price risks, that is, how spatial spillovers of public health scares to meat price risks vary depending on the increase in geographic distance. Public health scares over zoonotic disease may spread and diffuse across the regions, and local public health scares over zoonotic disease could spill over to its neighboring areas, leading to neighboring meat price risks. When the cross-regional effects of local public health scares on neighboring meat price risks exist at very short distances, a neighboring consumer could pick up a higher perceived risk of local public health scares over zoonotic disease and, thus, the spatial spillovers of local public health scares to neighboring meat price risks are quantitatively more significant in general; and when the geographic distance between local and neighboring areas increases, a neighboring consumer could pick up a lower perceived risk of local public health scares over zoonotic disease and, thus, the spatial spillovers of local public health scares to neighboring meat price risks may attenuate with the increase in geographic distance between local and neighboring areas. The existence of spatial attenuation boundaries suggests low interregional spatial market integration (as measured by deviations from the law of one price (LOP)) in meat products, i.e., inter-provincial meat market fragmentation.

Since recent research has provided evidence that spatial spillovers of public health scares to meat price risks might be complex and heterogeneous [37], we can infer that spatial spillovers of public health scares to meat price risks may not be monotone decreasing with the increase in geographic distance, but heterogeneous attenuating: When areas are geographically close, the clustered local public health scares over zoonotic disease have increasingly stronger regional spillovers; and when areas are geographically distant, spatial spillovers of public health scares to meat price risks attenuate. In other words, (i) if the geographic distance is below a certain threshold, the spatial spillovers of local public health scares to neighboring meat price risks may strengthen progressively with the increase in geographic distance; and (ii) if the geographic distance is above a certain threshold, the spatial spillovers of local public health scares to neighboring meat price risks may weaken progressively with the increase in geographic distance. A possible explanation for this is that (i) on the left side of the turning point (within mean provincial boundaries), the closer the meat markets to the epicenters of health scares, the stronger the effects of local government control, and the weaker the price impacts of health scares, and vice versa; hence, on the left side, local government control effects might partially counteract price impacts of public health scares; (ii) on the right side of the turning point (outside mean provincial boundaries), effects of local government control are comparatively weak, and price impacts of public health scares might surpass local government control effects. Thus, we propose Hypothesis H3.

**Hypothesis** **3** **(H3):** *Spatial spillovers of public health scares to meat price risks are distance-decaying with spatial attenuation boundaries. Spatial spillovers of public health scares over zoonotic disease to meat price risks decay with the increase in geographic distance, and distance-decaying spatial spillovers of public health scares to meat price risks are U-shaped, where there exist regional boundaries: (i) below a certain geographic distance threshold, spatial spillovers of public health scares to meat price risks strengthen with the increase in geographic distance; and (ii) above a certain geographic distance threshold, spatial spillovers of public health scares to meat price risks weaken with the increase in geographic distance.*

By explicitly distinguishing among high-risk, low-risk, and substitute meat, we further discuss distance effects on spatial spillovers of public health scares to meat price risks, in a setting with heterogeneous meat risk levels. Thus, we propose Hypothesis H4.

**Hypothesis** **4** **(H4):** *Distance effects on spatial spillovers of public health scares to meat price risks are heterogeneous across high-risk, low-risk, and substitute meat.*

**Figure 3:**
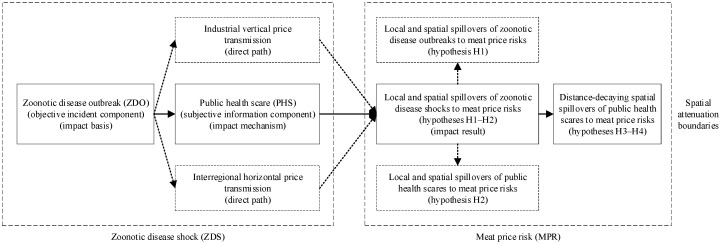
Analytical framework of hypotheses. The figure shows (i) that zoonotic disease outbreaks (ZDOs, objective incident component, impact basis) can either directly affect meat price risks (MPRs, impact result) through industrial vertical price transmission (direct path) or interregional horizontal price transmission (direct path), or indirectly affect meat price risks (MPRs, impact result) through public health scares (PHSs, subjective information component, impact mechanism); and (ii) local and spatial spillovers of zoonotic disease shocks to meat price risks (Hypotheses H1–H2) and distance-decaying spatial spillovers of public health scares to meat price risks (Hypotheses H3–H4). Source: Originally developed by the authors.

We employ an analytical framework to illustrate our hypotheses (Figure 3).

## 3. Materials and Methods

### 3.1. Data

The goal of our analysis is to identify the dynamic causal effects of zoonotic disease shocks on meat markets. Here we describe the research design that we use to overcome the endogeneity issues inherent in the identification of these effects. The ideal research design for identifying meat market responses to zoonotic disease shocks would randomly assign shocks to meat markets and track meat price risk responses over time. Moreover, such a research design would address the reverse causality concern. To achieve this, we utilize a research design that exploits the potential randomness of the timing of zoonotic disease shocks over a long period of time. Since highly pathogenic avian influenza (HPAI) shocks occur stochastically and spontaneously, they do not suffer from nonrandom assignment or reverse causality. Therefore, HPAI epidemics in meat markets can serve as exogenous shocks, facilitating the causal identification of spillovers of zoonotic disease shocks to meat price risks. Given the above, we employ HPAI epidemics in Chinese meat sector for 30 provinces with monthly observations from 2007 to 2017 as exogenous shocks and delve into the distance-decaying spillovers of zoonotic disease outbreaks and public health scares to meat price risks.

Our raw data come from six sources: (i) monthly meat prices from China Animal Agriculture Association (CAAA) (www.caaa.cn); (ii) Monthly human infection cases with HPAI (hand-collected) from Disease Surveillance (www.jbjc.org); (iii) monthly poultry infection cases with HPAI (hand-collected) from Official Veterinary Bulletin (www.cadc.net.cn); (iv) monthly Baidu search volumes (hand-collected) from Baidu Search (www.baidu.com); (v) monthly poultry production and consumption from EPS China Data (www.epschinadata.com); and (vi) province-level shapefiles from GADM data (gadm.org).

Using these sources, we construct a long spatial panel dataset for 30 provinces of mainland China (excluding Tibet) with monthly observations during November 2007–November 2017 (121 months), due to limited data availability.

Our raw data are processed as follows. (i) We address missing data using standard multiple imputation commands in Stata. (ii) We deflate all prices to real January 2004 RMBs. (iii) We take logs of continuous variables, winsorize them at the 1% level, and standardize them to have mean zero and unit variance, to eliminate outliers and facilitate interpretation.

### 3.2. Variables

First, meat price risk (MPR). We suppose that risk levels are heterogeneous across meat products, and distinguish among high-risk, low-risk, and substitute meat (as measured by live broiler, dressed broiler, and pork, respectively). This is because live broiler can be riskier to consumer than dressed broiler during HPAI epidemics, and pork could serve as a substitute for poultry products. Accordingly, we measure high-risk, low-risk, and substitute meat price risks using live broiler price, dressed broiler price, and pork price, respectively.

Second, zoonotic disease outbreak (ZDO). Since human infections with zoonotic diseases are more dangerous than animal infections, we differentiate between heterogeneous risk-level disease outbreaks, and use human and poultry infection cases with HPAI to measure high-risk and low-risk zoonotic disease cases. We hand-collect province-level panel data on human and poultry infection cases with HPAI from Disease Surveillance and Official Veterinary Bulletin, respectively, and convert the raw data into dummy variables, so as to better capture high-risk and low-risk zoonotic disease outbreaks.

Third, public health scare (PHS). We use Baidu search volume on HPAI to measure public health scare over zoonotic disease, due to the fact that (i) the internet becomes increasingly important over time and a major source of information spreading; (ii) online news and social media, which act as online opinion leaders, can detect salient information such as HPAI outbreaks, and amplify the information effects through two-step flow of communication, raising consumers’ risk perceptions of poultry products, thus inducing public health scares over HPAI and even mass consumers’ herding behavior towards health risks; and (iii) Baidu Search is the leading search engine in China, and Baidu search volume on HPAI can well represent the general public’s public health scares over HPAI. For this reason, we hand-collect province-level panel data on Baidu search volume on HPAI from Baidu Search, using the keywords “HPAI” and province names (e.g., “Beijing”, “Tianjin”, …, “Xinjiang”) (all in Chinese).

Fourth, price control variables. We include a vector of price control variables throughout the poultry supply chain (i.e., poultry feed price, poultry chick price, live poultry price, dressed poultry price, and pork price), to capture industrial vertical meat price transmission between upstream and downstream poultry sectors.

Fifth, supply and demand control variables. We also include several supply and demand control variables (i.e., aggregate poultry production, urban poultry consumption, and rural poultry consumption), to capture supply and demand in poultry sectors.

Sixth, spatial weighting matrices. We create two spatial weighting matrices (i.e., squared idistance matrix (*W*(1)) and exponential idistance matrix (*W*(2))) for spatial analysis, to capture interregional horizontal meat price transmission, that is, spatial spillovers of zoonotic disease shocks to meat price risks across locations. We use *W*(1) (Equation (1)) for our main spatial analysis, and *W*(2) (Equation (2)) for robustness checks.

(1) $$W(1):w_{ij}^{s}=\left\{ \begin{array}{l} \frac{1}{d_{ij}{}^{2}},i\neq j\\ 0,i=j \end{array}\right.\;w_{ij}^{{}^{\prime }s}=\left\{ \begin{array}{l} \frac{w_{ij}^{s}}{\sum_{j=1}^{n}w_{ij}^{s}},i\neq j\\ 0,i=j \end{array}\right.$$

(2) $$W(2):w_{ij}^{e}=\left\{ \begin{array}{l} \frac{1}{e^{d_{ij}/d_{\min}}},i\neq j\\ 0,i=j \end{array}\right.\;w_{ij}^{{}^{\prime }e}=\left\{ \begin{array}{l} \frac{w_{ij}^{e}}{\sum_{j=1}^{n}w_{ij}^{e}},i\neq j\\ 0,i=j \end{array}\right.$$

where *w^s^* indicates the spatial weight element associated to units *i* and *j* of squared inverse-distance spatial weighting matrix (*W*(1)); *w^e^* indicates the spatial weight element associated to units *i* and *j* of exponential inverse-distance spatial weighting matrix (*W*(2)); *w’^s^* and *w’^e^* indicate the row-normalizations; *d* indicates the geographical distance between the centroids of provinces *i* and *j*; and *d_min_* indicates the minimum geographic distance.

Table 1 provides a detailed description of data sources and variable definitions, and Table 2 provides summary statistics of the main variables after being processed.

### 3.3. Specifications of Hypotheses H1–H2

First, spillovers of zoonotic disease shocks to high-risk meat price risks. Since short-run spatial effects can only be characterized by dynamic spatial models, we develop dynamic spatial Durbin models (dynamic SDMs) and dynamic spatial autoregressive (dynamic SAR) models to capture the short- and long-run local and spatial spillovers of zoonotic disease shocks to high-risk meat price risks, following LeSage and Pace [43], Elhorst [44], and our previous work [36,37]. We begin with dynamic SDM for high-risk meat (Equation (3)):

(3) $$\begin{array}{c} \ln hrmpr_{it}=\ln\alpha +\tau \ln hrmpr_{it-1}+\psi \sum_{j=1}^{n}w_{ij}\ln hrmpr_{jt-1}+\rho \sum_{j=1}^{n}w_{ij}\ln hrmpr_{jt}\\ +\beta_{1}hrzdo_{it}+\beta_{2}lrzdo_{it}+\beta_{3}\ln phs_{it}+\beta_{4}X_{it}\\ +\theta_{1}\sum_{j=1}^{n}w_{ij}hrzdo_{it}+\theta_{2}\sum_{j=1}^{n}w_{ij}lrzdo_{it}+\theta_{3}\sum_{j=1}^{n}w_{ij}\ln phs_{it}+\gamma month_{t}+\mu_{i}+\varepsilon_{it} \end{array}$$

where ln*hrmpr*, *hrzdo*, *lrzdo*, and ln*phs* indicate high-risk meat price risk, high-risk zoonotic disease outbreak, low-risk zoonotic disease outbreak, and public health scare over zoonotic disease, respectively; *τ*, *ψ*, and *ρ* indicate temporally, spatiotemporally, and spatially lagged effects of dependent variable (high-risk meat price risk), respectively; *β* indicates main coefficients; *θ* indicates spatially lagged effects of key independent variables (high-risk zoonotic disease outbreak, low-risk zoonotic disease outbreak, and public health scare); *γ* and *μ* indicate linear monthly trend and province dummies, respectively; *ε* indicates an error term; and *X* is a vector of control variables (poultry feed price, poultry chick price, live poultry price, dressed poultry price, and pork price).

We then present dynamic SAR for high-risk meat (Equation (4)):

(4) $$\begin{array}{c} \ln hrmpr_{it}=\ln\alpha +\tau \ln hrmpr_{it-1}+\psi \sum_{j=1}^{n}w_{ij}\ln hrmpr_{jt-1}+\rho \sum_{j=1}^{n}w_{ij}\ln hrmpr_{jt}\\ +\beta_{1}hrzdo_{it}+\beta_{2}lrzdo_{it}+\beta_{3}\ln phs_{it}+\beta_{4}X_{it}+\gamma month_{t}+\mu_{i}+\varepsilon_{it} \end{array}$$

where *θ* (spatially lagged effects of key independent variables) is not included; and ln*hrmpr*, *hrzdo*, *lrzdo*, and ln*phs* indicate high–risk meat price risk, high-risk zoonotic disease outbreak, low-risk zoonotic disease outbreak, and public health scare over zoonotic disease, respectively.

Second, spillovers of zoonotic disease shocks to low-risk meat price risks. We replace ln*hrmpr* (high-risk meat price risk) with ln*lrmpr* (low-risk meat price risk) in Equations (3) and (4), and rewrite them as Equation (5) (dynamic SDM for low-risk meat) and Equation (6) (dynamic SAR for low-risk meat):

(5) $$\begin{array}{c} \ln lrmpr_{it}=\ln\alpha +\tau \ln lrmpr_{it-1}+\psi \sum_{j=1}^{n}w_{ij}\ln lrmpr_{jt-1}+\rho \sum_{j=1}^{n}w_{ij}\ln lrmpr_{jt}\\ +\beta_{1}hrzdo_{it}+\beta_{2}lrzdo_{it}+\beta_{3}\ln phs_{it}+\beta_{4}X_{it}\\ +\theta_{1}\sum_{j=1}^{n}w_{ij}hrzdo_{it}+\theta_{2}\sum_{j=1}^{n}w_{ij}lrzdo_{it}+\theta_{3}\sum_{j=1}^{n}w_{ij}\ln phs_{it}+\gamma month_{t}+\mu_{i}+\varepsilon_{it} \end{array}$$

(6) $$\begin{array}{c} \ln lrmpr_{it}=\ln\alpha +\tau \ln lrmpr_{it-1}+\psi \sum_{j=1}^{n}w_{ij}\ln lrmpr_{jt-1}+\rho \sum_{j=1}^{n}w_{ij}\ln lrmpr_{jt}\\ +\beta_{1}hrzdo_{it}+\beta_{2}lrzdo_{it}+\beta_{3}\ln phs_{it}+\beta_{4}X_{it}+\gamma month_{t}+\mu_{i}+\varepsilon_{it} \end{array}$$

where ln*lrmpr*, *hrzdo*, *lrzdo*, and ln*phs* indicate low-risk meat price risk, high-risk zoonotic disease outbreak, low-risk zoonotic disease outbreak, and public health scare over zoonotic disease, respectively.

Third, spillovers of zoonotic disease shocks to substitute meat price risks. We replace ln*hrmpr* (high-risk meat price risk) with ln*smpr* (substitute meat price risk) in Equations (3) and (4), and rewrite them as Equation (7) (dynamic SDM for substitute meat) and Equation (8) (dynamic SAR for substitute meat):

(7) $$\begin{array}{c} \ln smpr_{it}=\ln\alpha +\tau \ln smpr_{it-1}+\psi \sum_{j=1}^{n}w_{ij}\ln smpr_{jt-1}+\rho \sum_{j=1}^{n}w_{ij}\ln smpr_{jt}\\ +\beta_{1}hrzdo_{it}+\beta_{2}lrzdo_{it}+\beta_{3}\ln phs_{it}+\beta_{4}X_{it}\\ +\theta_{1}\sum_{j=1}^{n}w_{ij}hrzdo_{it}+\theta_{2}\sum_{j=1}^{n}w_{ij}lrzdo_{it}+\theta_{3}\sum_{j=1}^{n}w_{ij}\ln phs_{it}+\gamma month_{t}+\mu_{i}+\varepsilon_{it} \end{array}$$

(8) $$\begin{array}{c} \ln smpr_{it}=\ln\alpha +\tau \ln smpr_{it-1}+\psi \sum_{j=1}^{n}w_{ij}\ln smpr_{jt-1}+\rho \sum_{j=1}^{n}w_{ij}\ln smpr_{jt}\\ +\beta_{1}hrzdo_{it}+\beta_{2}lrzdo_{it}+\beta_{3}\ln phs_{it}+\beta_{4}X_{it}+\gamma month_{t}+\mu_{i}+\varepsilon_{it} \end{array}$$

where ln*smpr*, *hrzdo*, *lrzdo*, and ln*phs* indicate substitute meat price risk, high-risk zoonotic disease outbreak, low–risk zoonotic disease outbreak, and public health scare over zoonotic disease, respectively.

### 3.4. Specifications of Hypotheses H3–H4

First, distance-varying spatial weighting matrix. The above empirical analysis does not allow for the distance-decaying spatial spillovers of public health scares over zoonotic disease to meat price risks. Following recent literature (e.g., Halpern and Murakozy [38]; Yu, Liu, and Xuan [40]), we further investigate distance-decaying spatial attenuation boundaries of spatial spillovers of public health scares to meat price risks. To explore the distance-dependency of spatial spillovers of public health scares to meat price risks, we specify [*d_min_*, *d_max_*] as the interval for geographic distance, *d_min_* as the lower bound of the interval, and *d_max_* as the upper bound of the interval. We construct spatial spillover measures weighted by a function of distance, which is given by:

(9) $$W_{d}^{v}|d=d_{min},d_{min}+\tau ,d_{min}+2\tau ,\ldots \ldots ,d_{max}$$

where $W_{d}^{v}|d$ is a function of geographic distance, *d* is a geographic distance threshold, *d_min_* is the minimum geographic distance, *d_max_* is the maximum geographic distance, and *τ* is an incremental value of geographic distance from *d_min_* to *d_max_*, which we define as 20 km.

Using Equations (1), (2), and (9), we construct a distance-varying spatial weighting matrix:

(10) $$W_{d}^{v}={\left(w_{ij,d}^{v}\right)}_{N\times N}$$

where $W_{d}^{v}$ is a distance-varying spatial weighting matrix weighted by distance (Equation (9)).

Here we incorporate *W*(1) (Equation (1)) and $W_{d}^{v}|d$ (Equation (9)) into $W_{d}^{v}$ (Equation (10)), and rewrite Equation (10) as the distance-varying spatial weighting matrix (squared idistance), $W_{d}^{v(s)}$:

(11) $$W_{d}^{v(s)}:w_{ij,d}^{v(s)}=\left\{ \begin{array}{c} \frac{1}{d_{ij}{}^{2}},i\neq j,d_{ij}\geq d\\ 0,i\neq j,d_{ij}<d\\ 0,i=j \end{array}\right.\;w_{ij,d}^{{}^{\prime }v(s)}=\left\{ \begin{array}{c} \frac{w_{ij,d}^{v(s)}}{\sum_{j=1}^{n}w_{ij,d}^{v(s)}},i\neq j,d_{ij}\geq d\\ 0,i\neq j,d_{ij}<d\\ 0,i=j \end{array}\right.$$

where *w^v(s)^* indicates the spatial weight element associated to units *i* and *j* of distance-varying spatial weighting matrix (squared inverse-distance); *w’^v(s)^* indicates the row-normalization; and *d* indicates the geographical distance between the centroids of provinces *i* and *j*.

For robustness, we incorporate *W*(2) (Equation (2)) and $W_{d}^{v}|d$ (Equation (9)) into $W_{d}^{v}$ (Equation (10)), and rewrite Equation (10) as the distance-varying spatial weighting matrix (exponential idistance), $W_{d}^{v(e)}$:

(12) $$W_{d}^{v(e)}:w_{ij,d}^{v(e)}=\left\{ \begin{array}{c} \frac{1}{e^{d_{ij}/d_{\min}}},i\neq j,d_{ij}\geq d\\ 0,i\neq j,d_{ij}<d\\ 0,i=j \end{array}\right.\;w_{ij,d}^{{}^{\prime }v(e)}=\left\{ \begin{array}{l} \frac{w_{ij,d}^{v(e)}}{\sum_{j=1}^{n}w_{ij,d}^{v(e)}},i\neq j,d_{ij}\geq d\\ 0,i\neq j,d_{ij}<d\\ 0,i=j \end{array}\right.$$

where *w^v(e)^* indicates the spatial weight element associated to units *i* and *j* of distance-varying spatial weighting matrix (exponential inverse-distance); *w’^v(e)^* indicates the row-normalization; *d* indicates the geographical distance between the centroids of provinces *i* and *j*; and *d_min_* indicates the minimum geographic distance.

Second, spatial spillover measures (high-risk meat). (i) To calibrate the radiation radius of spatial spillovers of public health scares among neighboring areas, we define a distance threshold variable, *d*. (ii) We delete the areas within threshold *d*, from the distance-varying spatial weighting matrix (squared idistance), $W_{d}^{v(s)}$, to depict the distance-decaying spatial spillovers of public health scares to meat price risks, that is, whether or how spatial spillovers of public health scares to meat price risks decay with the increase in geographic distance. (iii) Since the shortest distance among Chinese provincial capitals is 96.07 km (between Beijing and Tianjin), we set 80 km as the initial threshold (i.e., *d_min_* = 80), use 20 km as the incremental value (i.e., *τ* = 20), and run our regressions of high-risk meat price risk 147 times from 80 km to 3000 km (i.e., $W_{d}^{v}|d$ = 80, 100, 120, …, 3000), implementing our benchmark spatial model (in this case dynamic SAR for high-risk meat, i.e., Equation (4)) with the distance-varying spatial weighting matrix (squared idistance), $W_{d}^{v(s)}$. (iv) We record spatial spillover measures for high-risk meat (i.e., short-run spatial spillover effects of public health scares on high-risk meat price risks, at different distance thresholds) and accompanying *p*-values. (v) Considering that there exist only few provinces in $W_{d}^{v(s)}$ when distance thresholds exceed 3000 km, and that spatial spillover effects of public health scares are volatile with considerable noise due to extreme outliers, we only accept the results with thresholds shorter than 3000 km.

Third, spatial spillover measures (low-risk meat). (i) We replace high-risk meat with low-risk meat, and run our regressions of low-risk meat price risk 147 times from 80 km to 3000 km, implementing our benchmark spatial model (in this case dynamic SAR for low-risk meat, i.e., Equation (6)) with the distance-varying spatial weighting matrix (squared idistance), $W_{d}^{v(s)}$. (ii) We record spatial spillover measures for low-risk meat (i.e., short-run spatial spillover effects of public health scares on low-risk meat price risks, at different distance thresholds) and accompanying *p*-values, with thresholds between 80 km and 300 km.

Fourth, spatial spillover measures (substitute meat). Since spatial spillovers of public health scares to substitute meat price risks are statistically insignificant, we record no spatial spillover measure for substitute meat.

Fifth, spatial spillover measures (robustness). For robustness checks, we repeat the procedure described above using $W_{d}^{v(s)}$ (squared idistance) instead of $W_{d}^{v(e)}$ (exponential idistance), and record spatial spillover measures for high- and low-risk meat with distance-varying spatial weighting matrix (exponential idistance).

## 4. Results

### 4.1. Diagnostic Tests

First, unit-root tests. Table 3 reports formal panel-data unit-root tests (including LLC, IPS, and Fisher-ADF tests) that reject unit root hypothesis at standard significance levels, which alleviates potential concerns about spurious correlation in our long panel data spanning 121 months and 30 provinces.

Second, Moran test for spatial dependence. We conduct the Moran test for potential spatial correlation in high-risk, low-risk, and substitute meat price risks spanning November 2007–November 2017 to see whether spatial variables should be incorporated, as reported in Table 4. Moran’s *Is* for high-risk, low-risk, and substitute meat (as defined by H_MI, L_MI, and S_MI, respectively), which are significant at 10% or better are almost all positive. This indicates that meat price risks are mostly positively spatially correlated across provinces, and spatially lagged effects of meat price risks should not be ignored.

Table 4 also shows that Moran’s *Is* fluctuate during the sample period of November 2007–November 2017 for different meat products. Among them, Moran’s *Is* for low-risk meat have the highest mean value (mean = 0.1203) and highest standard deviation (std. dev. = 0.1631), suggesting that low-risk meat price risks (as measured by log dressed broiler price) are the most volatile of the three series, and are the most spatially correlated across provinces; while Moran’s *Is* for substitute meat have the lowest mean value (mean = 0.0320) and lowest standard deviation (std. dev. = 0.1281), suggesting that substitute meat price risks (as measured by log pork price) are the least volatile of the three series, and are the least spatially correlated across provinces.

**Figure 4:**
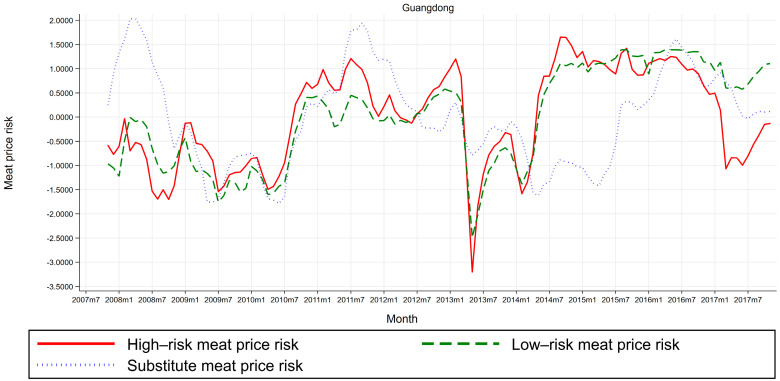
Line plots for time-series data for meat price risks in Guangdong. High-risk meat price risks, low-risk meat price risks, and substitute meat price risks are measured by log live broiler prices, log dressed broiler prices, and log pork prices, respectively. Source: Authors’ original calculations using Stata.

**Figure 5:**
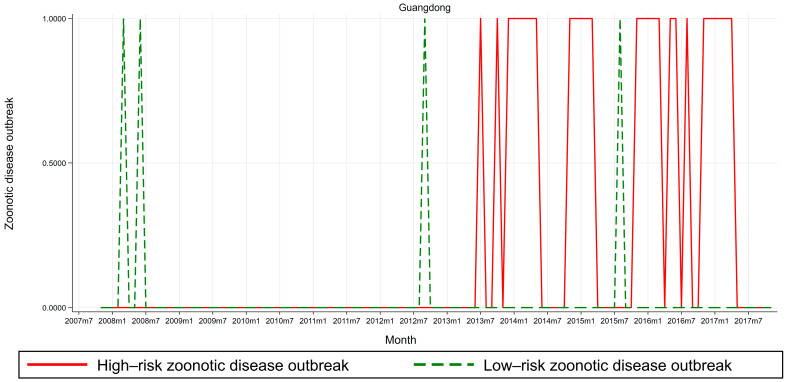
Line plots for time-series data for zoonotic disease outbreaks in Guangdong. High-risk zoonotic disease outbreaks and low-risk zoonotic disease outbreaks are measured by human infections with highly pathogenic avian influenza (HPAI) outbreak dummy and poultry infections with HPAI outbreak dummy, respectively. Source: Authors’ original calculations using Stata.

Third, risk-level heterogeneity tests. To present preliminary visual evidence on potential heterogeneous risk levels in meat price risks and zoonotic disease outbreaks, we plot trends in high-risk, low-risk, and substitute meat price risks in Figure 4, and trends in high-risk and low-risk zoonotic disease outbreaks in Figure 5, for a typical area where outbreaks of poultry infectious disease epidemics are more prevalent. We choose Guangdong since it is a HPAI epidemic-prone province [45,46]. Figure 4 and Figure 5 display line plots of meat price risks (high-risk, low-risk, and substitute) and zoonotic disease outbreaks (high-risk and low-risk), respectively, for time-series data for Guangdong province spanning the 121-month period from November 2007 to November 2017. (i) Line plots for meat price risks. Figure 4 shows that for Guangdong province, trends in high-risk meat price risks (as measured by live broiler prices) and low-risk meat price risks (as measured by dressed broiler prices) seem synchronized, whereas the trend in high-risk meat price risks is more volatile than its counterpart, in that the amplitude of high-risk meat price risks far exceeds that of low-risk meat price risks. Moreover, trend in substitute meat price risks (as measured by pork prices) does not synchronize with trends in high-risk and low-risk meat price risks. Thus, it is essential to differentiate among high-risk, low-risk, and substitute meat price risks, due to potential heterogeneous risk levels in meat price risks across the sample. (ii) Line plots for zoonotic disease outbreaks. Figure 5 shows that for Guangdong province, trends in high-risk zoonotic disease outbreaks (as measured by human infections with avian influenza outbreak dummy) and low-risk zoonotic disease outbreaks (as measured by poultry infections with avian influenza outbreak dummy) are asynchronous, which implies that high-risk and low-risk zoonotic disease outbreaks may not necessarily exist or occur at the same time. Thus, it is essential to differentiate between high- and low-risk zoonotic disease outbreaks, due to potential heterogeneous risk levels in zoonotic disease outbreaks across the sample.

### 4.2. Tests of Hypotheses H1–H2

First, benchmark estimates. Table 5 presents benchmark analysis of local and spatial spillovers of zoonotic disease shocks to meat price risks (Hypotheses H1–H2) using QML estimator. Columns (1)–(6) report estimates of Equation (3) (spillovers of zoonotic disease shocks to high-risk meat price risks using dynamic SDM), Equation (4) (spillovers of zoonotic disease shocks to high-risk meat price risks using dynamic SAR), Equation (5) (spillovers of zoonotic disease shocks to low-risk meat price risks using dynamic SDM), Equation (6) (spillovers of zoonotic disease shocks to low-risk meat price risks using dynamic SAR), Equation (7) (spillovers of zoonotic disease shocks to substitute meat price risks using dynamic SDM), and Equation (8) (spillovers of zoonotic disease shocks to substitute meat price risks using dynamic SAR), respectively.

Second, model selection. (i) All spatially lagged effects of dependent variables (ρ) are significant, so spatial models better fit the data than nonspatial models. (ii) BIC suggests that dynamic SAR has a better fit than dynamic SDM. (iii) Thus, we choose dynamic SAR as our benchmark spatial model (columns (2), (4), and (6)).

Third, spillovers of zoonotic disease shocks to high-risk meat price risks (column (2)). (i) In the short run, local and spatial spillovers of zoonotic disease outbreaks and public health scares to high-risk meat price risks are all significantly negative. (ii) In the long run, none of the spillovers are significant.

Fourth, spillovers of zoonotic disease shocks to low-risk meat price risks (column (4)). (i) In the short run, local and spatial spillovers of public health scares to low-risk meat price risks are both significantly negative, while none of the spillovers of zoonotic disease outbreaks to low-risk meat price risks are significant. (ii) In the long run, only local spillovers of public health scares to low-risk meat price risks are significantly negative, while others are all insignificant.

Fifth, spillovers of zoonotic disease shocks to substitute meat price risks (column (6)). (i) In the short run, only local spillovers of public health scares to substitute meat price risks are significantly negative, while others are all insignificant. (ii) In the long run, none of the spillovers are significant.

To recap, zoonotic disease outbreaks and public health scares have negative local and spatial spillovers to meat price risks, which are heterogeneous across high-risk, low-risk, and substitute meat. (i) For high-risk meat, short-run local and spatial spillovers of zoonotic disease outbreaks and public health scares to high-risk meat price risks are all significantly negative. (ii) For low-risk meat, short-run local and spatial spillovers of public health scares to low-risk meat price risks are both significantly negative, while none of the short-run spillovers of zoonotic disease outbreaks to low-risk meat price risks are significant. (iii) For substitute meat, only short-run local spillovers of public health scares to substitute meat price risks are significantly negative, while other short-run spillovers are all insignificant. (iv) In the long run, only long-run local spillovers of public health scares to low-risk meat price risks are significantly negative, while other long-run spillovers are all insignificant.

### 4.3. Tests of Hypotheses H3–H4

First, further estimates. Table 6 presents further analysis of distance-decaying spillovers of public health scares to meat price risks (Hypotheses H3–H4). Since model selection in benchmark analysis suggests that dynamic SAR best fits the data, we choose to use dynamic SAR. We incorporate $W_{d}^{v(s)}$ into dynamic SAR for high-risk meat (Equation (4)) and dynamic SAR for low-risk meat (Equation (6)), respectively, and obtain distance-varying dynamic SAR for high- and low-risk meat. We estimate distance effects on spatial spillover measures 147 times each for high-risk meat and low-risk meat, with 80 km as the initial threshold and 20 km as the incremental value, from 80 km to 3000 km, using distance-varying spatial weighting matrix (squared idistance), $W_{d}^{v(s)}$. As spatial spillovers of public health scares to substitute meat price risks are statistically insignificant (in line with our benchmark analysis), we record no spatial spillover measure for substitute meat. All spatial spillover measures are winsorized at the 3% level to reduce the possible effects of outliers.

Second, spatial spillover measures. Estimates indicate that long-run spatial spillover effects of public health scares on meat price risks are generally insignificant at the 10% level, with thresholds between 80 km and 300 km. This coincides with our benchmark estimates where distance effects are not accounted for. Our estimates also indicate that short-run spatial spillover effects of public health scares on meat price risks are generally significant at the 0.1% level, with thresholds between 80 km and 300 km. This suggests that distance effects on short-run spatial spillover measures are precisely estimated. Hence, spatial spillover measures are only recorded for short-run spatial spillover effects of public health scares on meat price risks.

**Figure 6:**
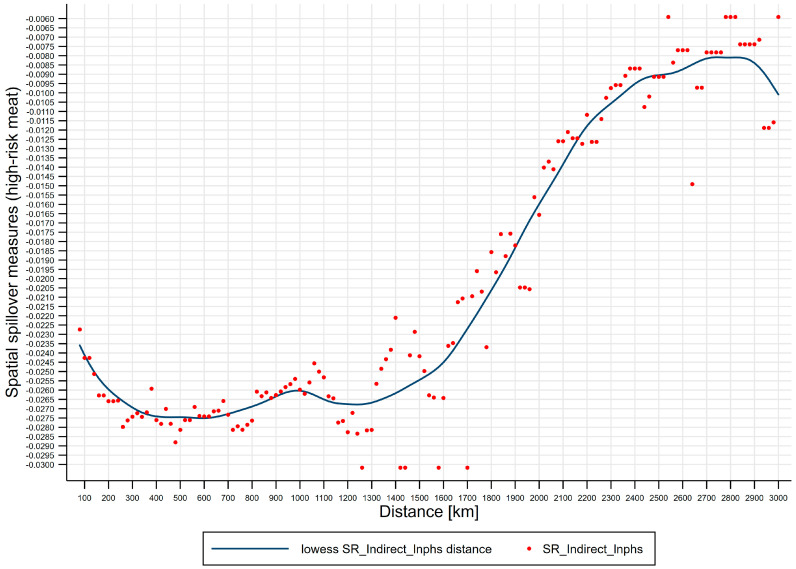
Distance-decaying spatial spillovers of public health scares to high-risk meat price risks, using distance-varying spatial weighting matrix (squared idistance), $W_{d}^{v(s)}$. Figure shows lowess line plot with scatterplot of distance effects on spatial spillover measures (high-risk meat) using 147 observations. The bandwidth (i.e., smoothing parameter) is specified as 0.2. Spatial spillover measures (high-risk meat) = short-run spatial spillover effects of public health scares on high-risk meat price risks, at different distance thresholds. Source: Authors’ original calculations using Stata.

Third, effects of distance on spatial spillover measures (high-risk meat). Figure 6 shows lowess line plot with scatterplot of distance effects on spatial spillover measures (high-risk meat). (i) The negative short-run spatial spillovers of public health scares to high-risk meat price risks are distance-varying, and short-run distance-decaying spatial spillovers of public health scares to high-risk meat price risks are U-shaped, where the distance turning point for high-risk meat is 500 km, which divides the U-shape into two regions: Region 1 [80, 500) the strengthening region and region 2 [500, 3000] the weakening region. We further subdivide the two regions into four regimes, i.e., spatial attenuation boundaries: Regime 1 [80, 500) the enhancing regime, regime 2 [500, 1400) the recovering regime, regime 3 [1400, 2200) the half-decaying regime, and regime 4 [2200, 3000] the slow-decaying regime, where regime 1 corresponds to region 1 and regimes 2–4 correspond to region 2. (ii) Regime 1 [80, 500) is the enhancing regime, spanning 420 km, from *d_min_* to the distance turning point for high-risk meat, where the negative short-run spatial spillovers enhance from −0.022 to −0.029. (iii) Regime 2 [500, 1400) is the recovering regime, spanning 900 km, from the distance turning point for high-risk meat to 1400 km, where the negative short-run spatial spillovers recover from −0.029 to −0.022. (iv) Regime 3 [1400, 2200) is the half-decaying regime, spanning 800 km, from 1400 km to 2200 km, where the negative short-run spatial spillovers quickly decay from −0.022 to −0.011. (v) Regime 4 [2200, 3000] is the slow-decaying regime, spanning 800 km, from 2200 km to 3000 km, where the negative short-run spatial spillovers slowly decay from −0.011 to −0.006.

**Figure 7:**
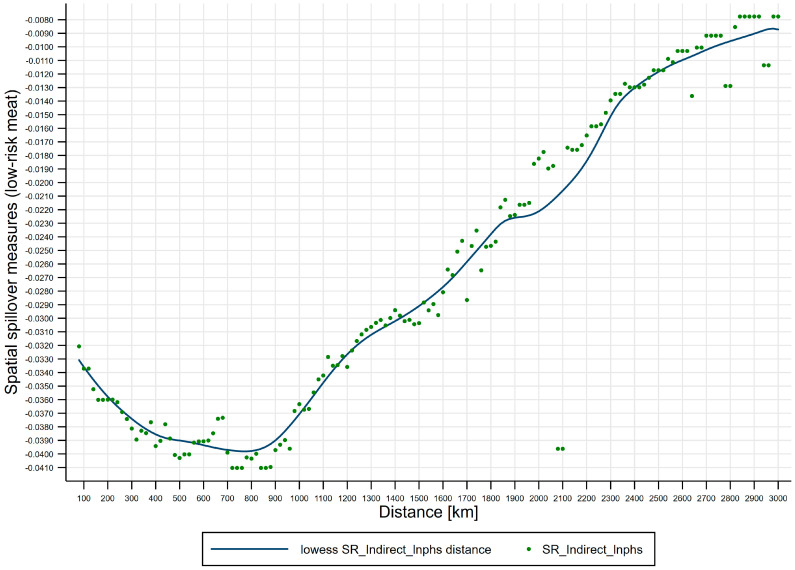
Distance-decaying spatial spillovers of public health scares to low-risk meat price risks, using distance-varying spatial weighting matrix (squared idistance), $W_{d}^{v(s)}$. Figure shows lowess line plot with scatterplot of distance effects on spatial spillover measures (low-risk meat) using 147 observations. The bandwidth (i.e., smoothing parameter) is specified as 0.2. Spatial spillover measures (low-risk meat) = short-run spatial spillover effects of public health scares on low-risk meat price risks, at different distance thresholds. Source: Authors’ original calculations using Stata.

Fourth, effects of distance on spatial spillover measures (low-risk meat). Figure 7 shows lowess line plot with scatterplot of distance effects on spatial spillover measures (low-risk meat). (i) The negative short-run spatial spillovers of public health scares to low-risk meat price risks are distance-varying, and short-run distance-decaying spatial spillovers of public health scares to low-risk meat price risks are U-shaped, where the distance turning point for low-risk meat is 800 km, which divides the U-shape into two regions: Region 1 [80, 800) the strengthening region and region 2 [800, 3000] the weakening region. We further subdivide the two regions into four regimes, i.e., spatial attenuation boundaries: Regime 1 [80, 800) the enhancing regime, regime 2 [800, 1200) the recovering regime, regime 3 [1200, 2200) the half-decaying regime, and regime 4 [2200, 3000] the slow-decaying regime, where regime 1 corresponds to region 1 and regimes 2–4 correspond to region 2. (ii) Regime 1 [80, 800) is the enhancing regime, spanning 720 km, from *d_min_* to the distance turning point for low-risk meat, where the negative short-run spatial spillovers enhance from −0.032 to −0.041. (iii) Regime 2 [800, 1200) is the recovering regime, spanning 400 km, from the distance turning point for low-risk meat to 1200 km, where the negative short-run spatial spillovers recover from −0.041 to −0.032. (iv) Regime 3 [1200, 2200) is the half-decaying regime, spanning 1000 km, from 1200 km to 2200 km, where the negative short-run spatial spillovers quickly decay from −0.032 to −0.016. (v) Regime 4 [2200, 3000] is the slow-decaying regime, spanning 800 km, from 2200 km to 3000 km, where the negative short-run spatial spillovers slowly decay from −0.016 to −0.008.

**Figure 8:**
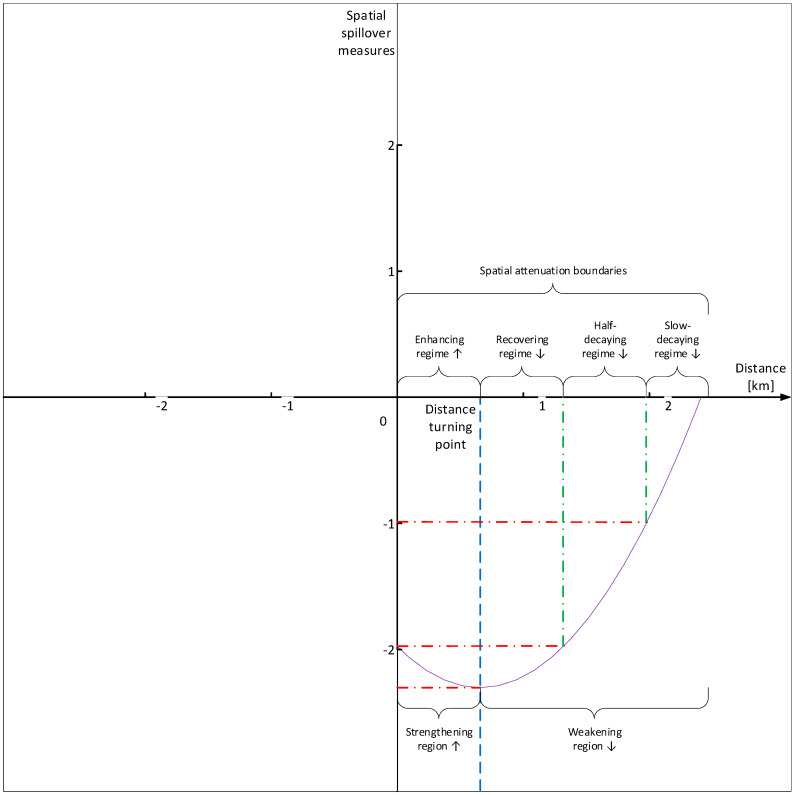
Graphical illustration of spatial attenuation boundaries. Figure shows distance effects on spatial spillover measures, which are heterogeneous in high-risk, low-risk, and substitute meat. Spatial spillover measures = short-run spatial spillover effects of public health scares on meat price risks, at different distance thresholds. Violet curve = effects of distance on spatial spillover measures. Dashed blue line = distance turning point. Dot-dashed green line = distance boundaries (i.e., spatial attenuation boundaries). Dot-dashed red line = spatial spillover measures boundaries. Source: Originally developed by the authors.

To recap, distance effects on spatial spillover measures are U-shaped with spatial attenuation boundaries and heterogeneous across high-risk, low-risk, and substitute meat (as illustrated in Figure 8). (i) For high-risk meat, distance turning point of the U-shape is 500 km, with four spatial attenuation boundaries, that is, enhancing regime [80, 500), recovering regime [500, 1400), half-decaying regime [1400, 2200), and slow-decaying regime [2200, 3000]. (ii) For low-risk meat, distance turning point of the U-shape is 800 km, with four spatial attenuation boundaries, that is, enhancing regime [80, 800), recovering regime [800, 1200), half-decaying regime [1200, 2200), and slow-decaying regime [2200, 3000]. (iii) For substitute meat, distance effects on spatial spillover measures are insignificant, with no spatial attenuation boundaries.

### 4.4. Robustness Checks

First, further estimates (robustness). Table 7 presents further analysis of distance-decaying spillovers of public health scares to meat price risks (Hypotheses H3–H4) (robustness). For robustness checks, we repeat the procedure described in Section 4.4 using $W_{d}^{v(s)}$ (squared idistance) instead of $W_{d}^{v(e)}$ (exponential idistance), and record spatial spillover measures for high- and low-risk meat with distance-varying spatial weighting matrix (exponential idistance). As spatial spillovers of public health scares to substitute meat price risks are statistically insignificant (in line with our benchmark analysis), we record no spatial spillover measure for substitute meat.

Second, spatial spillover measures. Estimates indicate that long-run spatial spillover effects of public health scares on meat price risks are generally insignificant at the 10% level, and short-run spatial spillover effects of public health scares on meat price risks are generally significant at the 0.1% level, with thresholds between 80 km and 300 km. This is consistent with our further estimates using distance-varying spatial weighting matrix (squared idistance), $W_{d}^{v(s)}$.

**Figure 9:**
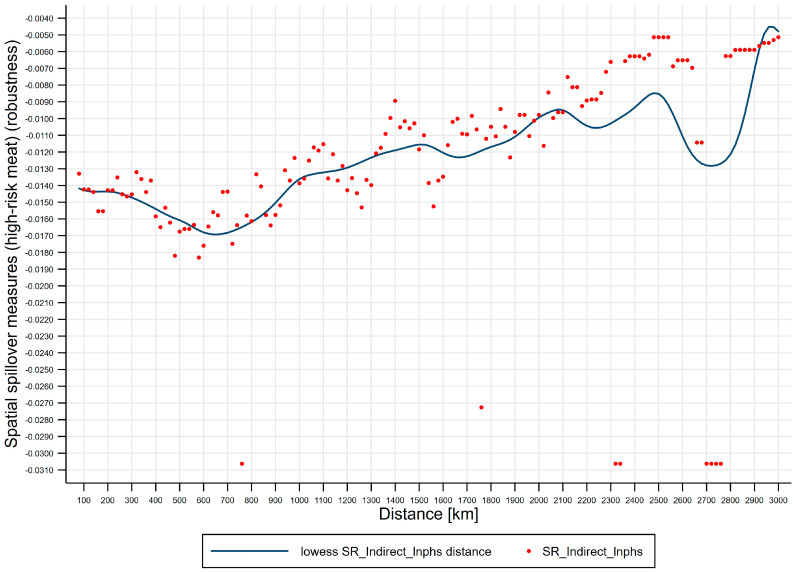
Distance-decaying spatial spillovers of public health scares to meat price risks (high-risk meat) (robustness), using distance-varying spatial weighting matrix (exponential idistance), $W_{d}^{v(e)}$. Figure shows lowess line plot with scatterplot of distance effects on spatial spillover measures (high-risk meat) using 147 observations. The bandwidth (i.e., smoothing parameter) is specified as 0.2. Spatial spillover measures (high-risk meat) = short-run spatial spillover effects of public health scares on high-risk meat price risks, at different distance thresholds. Source: Authors’ original calculations using Stata.

Third, effects of distance on spatial spillover measures (high-risk meat) (robustness). Figure 9 shows lowess line plot with scatterplot of distance effects on spatial spillover measures (high-risk meat) (robustness), using distance-varying spatial weighting matrix (exponential idistance), $W_{d}^{v(e)}$. The figure indicates that, for high-risk meat, distance effects on spatial spillover measures are U-shaped with similar spatial attenuation boundaries, and distance turning point of the U-shape is 600 km. This closely resembles our further estimates using distance-varying spatial weighting matrix (squared idistance), $W_{d}^{v(s)}$.

**Figure 10:**
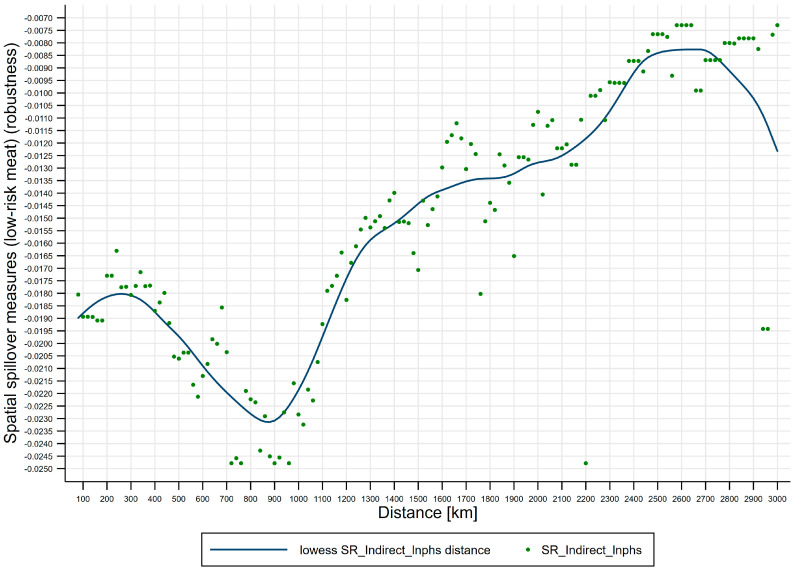
Distance-decaying spatial spillovers of public health scares to meat price risks (low-risk meat) (robustness), using distance-varying spatial weighting matrix (exponential idistance), $W_{d}^{v(e)}$. Figure shows lowess line plot with scatterplot of distance effects on spatial spillover measures (low-risk meat) using 147 observations. The bandwidth (i.e., smoothing parameter) is specified as 0.2. Spatial spillover measures (low-risk meat) = short-run spatial spillover effects of public health scares on low-risk meat price risks, at different distance thresholds. Source: Authors’ original calculations using Stata.

Fourth, effects of distance on spatial spillover measures (low-risk meat) (robustness). Figure 10 shows lowess line plot with scatterplot of distance effects on spatial spillover measures (low-risk meat) (robustness), using distance-varying spatial weighting matrix (exponential idistance), $W_{d}^{v(e)}$. Figure indicates that, for low-risk meat, distance effects on spatial spillover measures are U-shaped with similar spatial attenuation boundaries, and distance turning point of the U-shape is 900 km. This closely resembles our further estimates using distance-varying spatial weighting matrix (squared idistance), $W_{d}^{v(s)}$.

To recap, our results are robust to alternative spatial weighting matrices.

## 5. Discussion

### 5.1. Discussion on Hypotheses H1–H2

**Figure 11:**
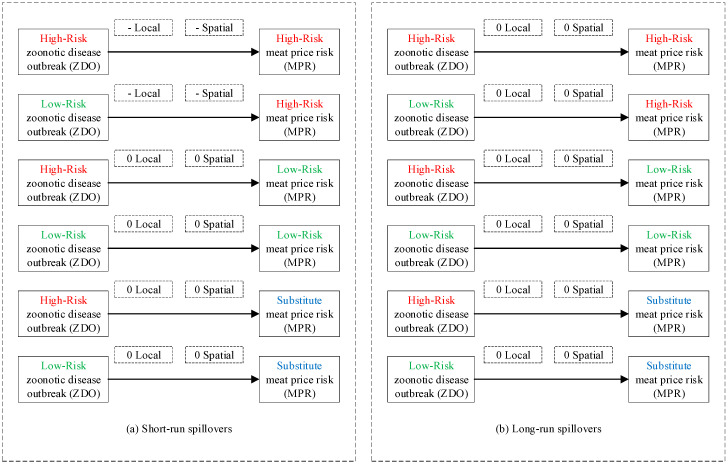
Conceptual diagram of local and spatial spillovers of zoonotic disease outbreaks to meat price risks (Hypothesis H1). The figure shows that (i) for high-risk meat, both high-risk and low-risk zoonotic disease outbreaks have negative local and spatial spillovers to meat price risks; (ii) for low-risk and substitute meat, neither high-risk nor low-risk zoonotic disease outbreaks have significant local or spatial spillovers to meat price risks; and (iii) in the long run, none of the spillovers are significant. The symbols “-” and “0” show “negative” and “insignificant” spillovers, respectively. Source: Originally developed by the authors.

**Figure 12:**
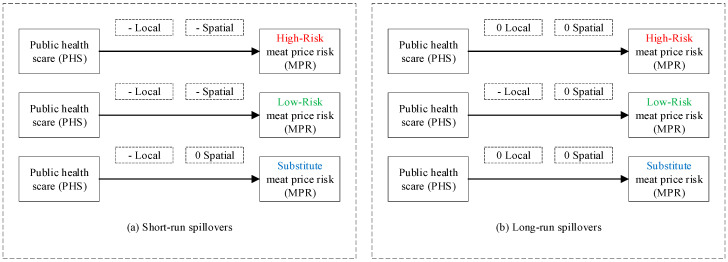
Conceptual diagram of local and spatial spillovers of public health scares to meat price risks (Hypothesis H2). The figure shows that (i) for high-risk and low-risk meat, public health scares over zoonotic disease have negative local and spatial spillovers to meat price risks; (ii) for substitute meat, public health scares over zoonotic disease only have negative local spillovers to meat price risks, while spatial spillovers of public health scares to meat price risks are statistically insignificant; and (iii) in the long run, only local spillovers of public health scares to low-risk meat price risks are significantly negative, while others are statistically insignificant. The symbols “-” and “0” show “negative” and “insignificant” spillovers, respectively. Source: Originally developed by the authors.

First, statements of results. The test results in Table 5 are consistent with Hypotheses H1–H2 that zoonotic disease outbreaks and public health scares have negative local and spatial spillovers to meat price risks, which are heterogeneous across high-risk, low-risk, and substitute meat. More specifically: (i) For high-risk meat, short-run local and spatial spillovers of zoonotic disease outbreaks and public health scares to high-risk meat price risks are all significantly negative. (ii) For low-risk meat, short-run local and spatial spillovers of public health scares to low-risk meat price risks are both significantly negative, while none of the short-run spillovers of zoonotic disease outbreaks to low-risk meat price risks are significant. (iii) For substitute meat, only short-run local spillovers of public health scares to substitute meat price risks are significantly negative, while other short-run spillovers are all insignificant. (iv) In the long run, only long-run local spillovers of public health scares to low-risk meat price risks are significantly negative, while other long-run spillovers are all insignificant. We illustrate test results and conclusions for Hypotheses H3–H4 in Figure 11 and Figure 12, respectively.

Second, relation with previous literature. (i) Reference to previous research on spillovers of zoonotic disease outbreaks to meat price risks. Although a growing literature analyzes the impacts of zoonotic disease outbreaks on food prices, previous literature typically focuses on the direct effects of zoonotic disease outbreaks on their corresponding animal product price levels, without accounting for potential spillover effects across regions; in addition, potential risk-level heterogeneity in zoonotic disease outbreaks and food products are not considered [11,19,20,24]. However, we set out to identify both the local and spatial spillover effects of zoonotic disease outbreaks on meat price risks using dynamic spatial models (i.e., dynamic SDM and dynamic SAR), by distinguishing among high-risk, low-risk, and substitute meat products and between high-risk and low-risk zoonotic disease outbreaks, and suggest that zoonotic disease outbreaks themselves only induce high-risk meat price risks, not low-risk or substitute meat price risks. Therefore, our finding complements our knowledge on the spillover effects of zoonotic disease outbreaks on meat price risks with risk-level heterogeneity, which is obscured in prior literature.

(ii) Reference to previous research on spillovers of public health scares to meat price risks. Although a large body of literature examines the impacts of food scares on food prices, previous literature typically assesses the total effects of zoonotic disease shocks, not public health scares alone, on related animal product prices, regardless of the potential components of zoonotic disease shocks; also, food price volatility and transmission, rather than food price risk spillovers, are the main focus of the literature, where time series models are usually used [12,13,21,22,23,25]. However, we differentiate between the components of zoonotic disease shocks by decomposing zoonotic disease shocks into zoonotic disease outbreaks (objective incident component) and public health scares (subjective information component), so as to quantify local and spatial spillovers of public health scares to meat price risks by employing dynamic spatial panel data models, and suggest that public health scares magnify negative meat price risks induced by zoonotic disease outbreaks. Therefore, our finding elucidates the impact mechanism (i.e., public health scares) by which zoonotic disease outbreaks exert their influences on meat price risks in a setting with meat-specific risk levels, which contributes to our understanding of the underlying economic mechanisms.

Third, interpretations of results. (i) Interpretations of results on spillovers of zoonotic disease outbreaks to meat price risks. When zoonotic diseases occur, they may affect the whole supply chain of related meat sector [7,47,48]. For instance, HPAI outbreaks can directly impact the supply side of poultry industry, causing poultry infections, deaths, and destruction implemented by authorities, which lowers poultry production [49,50]. Besides, HPAI outbreaks might impact poultry demand side, by raising consumers’ risk perceptions of poultry products [51,52]. For high-risk poultry products such as live broiler, consumers may considerably reduce purchase volumes, for fear of getting infected with HPAI; and live broiler demand reduction could dramatically exceed supply reduction, thereby likely putting downward pressure on high-risk poultry prices. Furthermore, the price risks of high-risk poultry products could even spill over to nearby poultry markets by interregional horizontal price transmission mechanism, because of food spatial market integration (i.e., the law of one price (LOP) across markets). On the other hand, for low-risk poultry products such as dressed broiler, dressed broiler supply and demand reductions might roughly offset each other, thus rendering low-risk poultry price risks insignificant. Nonetheless, in the long term, poultry markets can converge to equilibrium prices after the transitory HPAI shocks, as a consequence of slow poultry demand recoveries and gradual poultry price reversals.

(ii) Interpretations of results on spillovers of public health scares to meat price risks. With the advent of big data, online media (such as online news and social media) have had profound impacts on consumers’ food risk perceptions through herding. To illustrate, as highly available and salient information [53,54], HPAI outbreaks could attract high online media attention, leading to a surge in online media coverage of the zoonotic disease incidents. Excessive online media coverage of the outbreaks might not only boost individual consumers’ risk perceptions of related poultry products, but could also give rise to mass consumers’ excessive attention to the potential food risks, owing to online media effects, from online press coverage associated with the disease outbreaks and online social interactions across consumers. That would fuel mass consumers’ HPAI food scares, namely, general public’s health scares over HPAI. This, in turn, may exacerbate mass consumers’ herding behavior towards health risks, resulting in a collapse in poultry demand. In response, poultry prices can decline sharply very shortly after online media coverage of HPAI. Since online information spreads rapidly across individuals and even regions through online news and social media, general public’s health scares over HPAI could transmit to nearby locations and cause poultry price risks in neighboring markets. Moreover, local prices of substitute meat for poultry products (such as pork) may also be affected by HPAI health scares, on account of industrial vertical meat price transmission mechanism. While high-risk poultry products (such as live broiler) converge to equilibrium prices in the long term, low-risk poultry products (such as dressed broiler) do not. This may be because, in contrast to intermediate products (i.e., live broiler) in midstream poultry sector, consumer products (i.e., dressed broiler) in downstream poultry sector might be more susceptible to food demand shocks induced by HPAI health scares, thus resulting in long-run effects of HPAI health scares on low-risk poultry prices.

### 5.2. Discussion on Hypotheses H3–H4

**Figure 13:**
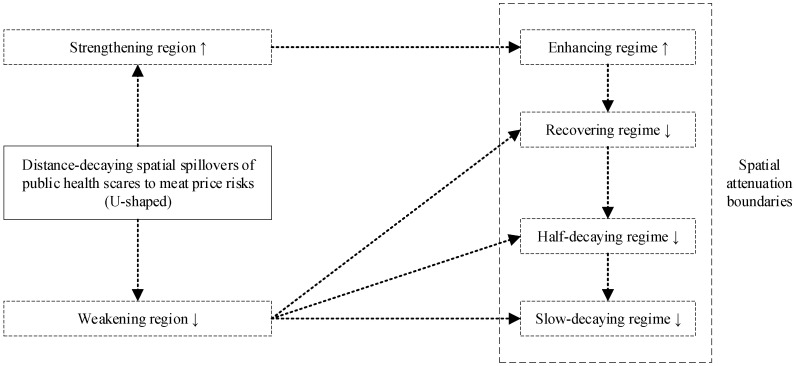
Conceptual diagram of distance-decaying spatial spillovers of public health scares to meat price risks (Hypotheses H3–H4). The figure shows that (i) the negative short-run spatial spillovers of public health scares over zoonotic disease to meat price risks are distance-decaying, and the short-run effects of distance on spatial spillovers of public health scares to meat price risks are U-shaped, where there exist distance-decaying spatial attenuation boundaries: The distance turning point divides the U-shape into the strengthening region and the weakening region, which are further subdivided into the enhancing regime, the recovering regime, the half-decaying regime, and the slow-decaying regime; (ii) the distance turning point is lower for high-risk meat (500 km) than it is for low-risk meat (800 km); and (iii) as spatial spillovers of public health scares to substitute meat price risks are statistically insignificant, there exist no distance-decaying spatial attenuation boundaries for substitute meat. Source: Originally developed by the authors.

First, statements of results. The test results in Table 6 and Figure 6 and Figure 7 are consistent with Hypotheses H3–H4 that the negative spatial spillovers of public health scares over zoonotic disease to meat price risks are distance-decaying, and the effects of distance on spatial spillovers of public health scares to meat price risks are U-shaped, where there exist distance-decaying spatial attenuation boundaries, which are heterogeneous across high-risk, low-risk, and substitute meat. Therefore, we conclude that spatial spillovers of public health scares over zoonotic disease to meat price risks decay with distance, where meat-specific spillovers first strengthen and then weaken. More specifically: (i) For high-risk meat, the distance turning point of the U-shape is 500 km, with four spatial attenuation boundaries, that is, enhancing regime [80, 500), recovering regime [500, 1400), half-decaying regime [1400, 2200), and slow-decaying regime [2200, 3000]; (ii) for low-risk meat, the distance turning point of the U-shape is 800 km, with four spatial attenuation boundaries, that is, enhancing regime [80, 800), recovering regime [800, 1200), half-decaying regime [1200, 2200), and slow-decaying regime [2200, 3000]; (iii) for substitute meat, distance effects on spatial spillover measures are insignificant, with no spatial attenuation boundaries. We illustrate test results and conclusions for Hypotheses H3–H4 in Figure 13.

Second, relation with previous literature on distance-decaying spillovers of public health scares to meat price risks. Although prior literature explores the distance-decaying features of food prices, previous literature typically analyzes the effects of geographical distance (i.e., transport costs) on food price levels, food price volatility, and food price transmission, so as to test if food spatial market integration as well as the law of one price (LOP) holds [26,27,28,29,30,31,32]. However, we seek to characterize the effects of distance on spatial spillovers of public health scares to meat price risks, by constructing spillover measures weighted by distance following Halpern and Murakozy [38], and by performing our distance-decaying dynamic SAR regressions 147 times, and suggest that distance effects on spatial spillovers of public health scares to meat price risks are U-shaped. Therefore, our finding unveils the distance-decaying spatial spillover effects of public health scares to meat price risks, which thus complements the extant literature and provides novel evidence.

Third, interpretations of results on distance-decaying spillovers of public health scares to meat price risks. Geographic distance is an important determinant of the spatial spillover effects of health scares on related food prices. As an illustration, the negative spatial spillovers of HPAI health scares to poultry prices decay with geographic distance, which first strengthen and then weaken.

We find that health scares have negative spatial spillover effects on poultry prices, and these effects are distance-decaying. A possible explanation for this might be that (i) geographic distance positively correlates with social distance, and nonstandardized information (i.e., health scares) decays with the increase in social distance related to interpersonal communications and social media coverage; (ii) administrative power dominates economic activities within the region, and administrative regional boundaries block interregional factor mobility and horizontal price transmission; and (iii) consumers pay more attention to health scares in local and neighboring areas, and have weaker risk perceptions when farther away from the epicenters of outbreaks.

Results suggest that, for high-risk poultry products (such as live broiler), the distance effects on spatial spillover measures are U-shaped with a distance turning point of 500 km. A possible explanation for this is that (i) mean Chinese provincial boundaries are approximately 500 km [40], and on the left side of the turning point (within mean provincial boundaries), the closer the poultry markets to the epicenters of health scares, the stronger the effects of local government control, and the weaker the price impacts of health scares, and vice versa; therefore, on the left side, local government control effects could partially offset price impacts of health scares; (ii) on the right side of the turning point (outside mean provincial boundaries), effects of local government control are relatively weak, and price impacts of health scares decay with the increase in distance; therefore, on the right side, price impacts of health scares could prevail over local government control effects; and (iii) the existence of spatial attenuation boundaries suggests low interregional spatial market integration [55] (as measured by deviations from the law of one price (LOP)) in poultry products, that is, inter-provincial poultry market fragmentation [56].

Results also indicate that, for low-risk poultry products (such as dressed broiler), distance effects on spatial spillover measures are U-shaped with a distance turning point of 800 km, which is longer than that for high-risk poultry products. This result may be explained by the fact that (i) in contrast to live broiler, an intermediate product in midstream poultry supply chain, dressed broiler is a consumer product in downstream poultry supply chain, and directly faces end-users, where effects of local government control are stronger than that for live broiler; further, these effects are reinforced by retail market power, and local government control effects and retail market power could partially offset price impacts of health scares jointly; therefore, price impacts of health scares on dressed broiler attain their maximum later than that on live broiler, and the distance turning point is longer for dressed broiler than it is for live broiler; (ii) consuming dressed broiler during the epidemic is safer than consuming live broiler, and thus consumers’ risk perceptions of dressed broiler are lower than that of live broiler; therefore, price responses of dressed broiler to health scares are slower than that of live broiler, and the distance turning point of dressed broiler is farther; and (iii) distance turning point value is higher for dressed broiler than for live broiler, suggesting that interregional spatial market integration in dressed broiler is higher.

### 5.3. Limitations

Our long spatial panel dataset for 30 provinces of mainland China (excluding Tibet) with monthly observations during November 2007–November 2017 (121 months) potentially suffers from a few limitations, however. First, for smaller countries, distance decay of health scare effects on food prices might be significant across nations, not across regions. (i) We document that consumers would pay more attention to health scares that occur in local and neighboring regions, and could have lower risk perceptions when farther away from the epicenters of outbreaks, since individuals living closer to the epicenters of outbreaks are relatively more likely to be concerned with being affected by the disease epidemics. This is true for big countries like China and India where territories are quite large and regions are not well integrated [57]. For these big countries, health scare effects on consumers’ food demand and food prices can be significantly distance-decaying across regions within nations. (ii) Yet, for smaller countries like some European countries where territories are not that large, consumers might pay relatively equally high attention to health scares that occur all around their countries, no matter how close consumers live to the epicenters of outbreaks. Therefore, for smaller countries, distance decay of health scare effects on consumers’ food demand and food prices might be insignificant across regions within nations. Meanwhile, consumers may also be concerned with health scares that occur in neighboring countries and could have higher risk perceptions when closer to the epicenters of outbreaks, since cross-border disease transmission might be relatively fast for some smaller countries. As a result, for smaller countries, health scare effects on consumers’ food demand and food prices might be distance-decaying across nations, not across regions within nations.

Second, we restrict attention to distance-decaying spatial spillovers in food markets, whereas food price volatility is not our main focus. In this paper, we propose and implement a novel spatial econometric approach to detect potential nonmonotonic distance decay of health scare effects on food prices, using dynamic spatial panel-data models. However, meat price volatility based on time series regressions is not our primary goal, and future work could extend our empirical research by accounting for the duration of consumers’ food demand deficiency [37], the dynamic effects of exogenous disease shocks to food prices, and the regime switches in food price volatility during epidemics.

## 6. Conclusions

Our analysis has yielded the following salient findings. Collectively, health scares caused by disease outbreaks negatively spill over to meat prices, with U-shaped distance-decaying spatial effects. We contribute to the literature on disease shocks to consumer markets by documenting nonmonotonic distance decay of health scare effects on food prices, previously not found by the literature.

First, disease outbreaks have negative impacts on meat prices in local and neighboring markets, especially for high-risk meat. Consistent with the literature, we find evidence that zoonotic disease outbreaks have negative local and spatial spillovers to meat price risks. In particular, we further find that zoonotic disease outbreaks only locally and spatially spill over to high-risk meat products and result in price risks, not to low-risk or substitute meat products. Nevertheless, no spillovers of zoonotic disease outbreaks to meat products are detected in the long run. Our finding is associated with previous literature claiming that disease outbreaks may affect the whole supply chain of related meat sector, by directly impacting the supply side of related meat sector, and by raising consumers’ risk perceptions of related meat products on the demand side.

Second, health scares have negative impacts on meat prices in local and neighboring markets, and spill over to substitute meat in local markets. In accordance with most of the literature, we show that public health scares have negative local and spatial spillovers to meat price risks. Furthermore, we document that public health scares locally and spatially spill over to both high- and low-risk meat products causing corresponding price risks, and even spill over to substitute meat products locally. Evidence also indicates negative local spillovers of public health scares to low-risk meat products in the long run. Our finding is consistent with prior research arguing that during epidemics, online media have had profound impacts on consumers’ meat risk perceptions through herding, which would fuel mass consumers’ health scares and result in a collapse in related meat demand.

Third, spatial spillovers of health scares to meat prices are U-shaped with respect to distances, and distance turning point is shorter for high-risk meat than for low-risk meat. We add to the previous literature by constructing spatial spillover measures weighted by geographic distance and argue that spatial spillovers of public health scares to meat price risks decay with the increase in distance, and turn out to be a U-shaped function of distance, which first strengthens and then weakens. We also identify four spatial attenuation boundaries in the U-shape, that is, enhancing regime, recovering regime, half-decaying regime, and slow-decaying regime. Moreover, evidence suggests that the spatial attenuation boundaries are heterogeneous across meat products, and high-risk meat has a lower distance turning point value than its counterpart. Our finding complements existing work by highlighting that health scare effects on food prices nonmonotonically decay with distances, which suggests low interregional spatial market integration in meat products, that is, inter-provincial meat market fragmentation, due to distance decay of nonstandardized information and local government control effects across provincial boundaries.

These findings have two important policy implications. (i) Our evidence suggests that health scares can exacerbate disease shocks to meat prices, and could even spatially spill over to neighboring markets and cause widespread price risks in related meat products; therefore, the state authorities should increase information transparency of disclosures of disease outbreaks during epidemics to mitigate information asymmetry between the general public and the government, and curb irrational rumor spreading of health scares transmitted and fueled by online unofficial news and social media. (ii) Our finding indicates that the spatial spillover effects of health scares on related meat prices are distance-decaying and U-shaped with respect to distances, with spatial attenuation boundaries, perhaps due to distance decay of rumor spreading and attenuation effects of local government control measures across provincial boundaries; hence, local authorities are encouraged to take measures to regulate irrational rumor spreading of health scares over wider areas around the epicenters of epidemics, so as to counteract the adverse impacts of health scares on regional meat markets, and to weaken the distance-decaying spatial effects at a faster pace.

Three future research avenues seem particularly promising. (i) Our econometric analysis relies on province-level spatial panel data, whereas a city-level or even county-level spatial panel dataset could provide a more precise identification of the spatial spillover effects. (ii) We measure high-risk, low-risk, and substitute meat price risks by related meat prices, respectively, whereas meat price variations in meat price volatility [58] likely provide better measures of meat price risks than meat price levels. (iii) We conduct our empirical tests using dynamic spatial Durbin and dynamic spatial autoregressive models, whereas one could draw spatial causal inferences by estimating the spatial difference-in-differences models [59] that account for spatial autocorrelations in meat price risks in the areas “before and after” zoonotic disease outbreaks, or by using a spatial regression discontinuity approach [60] to identify the average effects of public health scares on meat price risks close to the border, where the case for identification may become more convincing.

## Figures and Tables

**Table 1:** Data and variable definitions.

Category	Concept	Variable	Measurement	Indicator	Source
Dependent variable	MPR	ln*hrmpr*	High–risk meat price risk	Log live broiler price	CAAA
(meat price risk)		ln*lrmpr*	Low–risk meat price risk	Log dressed broiler price	CAAA
		ln*smpr*	Substitute meat price risk	Log pork price	CAAA
Key independent variable	ZDO	*hrzdo*	High–risk zoonotic disease outbreak	Human infection with HPAI outbreak dummy	Disease Surveillance
(zoonotic disease shock)		*lrzdo*	Low–risk zoonotic disease outbreak	Poultry infection with HPAI outbreak dummy	Official Veterinary Bulletin
	PHS	ln*phs*	Public health scare over zoonotic disease	Log Baidu search volume on HPAI	Baidu Search
Price control variable		ln*pfp*	Poultry feed price	Log broiler feed price	CAAA
(industrial vertical price transmission)		ln*pcp*	Poultry chick price	Log broiler chick price	CAAA
		ln*hrmpr*	Live poultry price	Log live broiler price	CAAA
		ln*lrmpr*	Dressed poultry price	Log dressed broiler price	CAAA
		ln*smpr*	Pork price	Log pork price	CAAA
Supply and demand control variable		ln*apo*	Aggregate poultry production	Log aggregate poultry production	EPS China Data
(meat supply and demand)		ln*upc*	Urban poultry consumption	Log urban poultry consumption	EPS China Data
		ln*rpc*	Rural poultry consumption	Log rural poultry consumption	EPS China Data
Spatial weighting matrix		*w^s^*	Squared idistance matrix	Squared inverse-distance spatial weighting matrix	GADM data
(interregional horizontal price transmission)		*w^e^*	Exponential idistance matrix (robustness)	Exponential inverse-distance spatial weighting matrix	GADM data

Source: All data are originally collected by the authors.

**Table 2:** Summary statistics.

Variable	Measurement	N	Mean	Std. dev.	p1	p5	p25	p50	p75	p95	p99
ln*hrmpr*	High–risk meat price risk	3630	0.0000	0.9960	−2.5262	−1.6744	−0.6609	0.1223	0.7346	1.3697	1.8826
ln*lrmpr*	Low–risk meat price risk	3630	0.0000	0.9960	−2.3163	−1.6338	−0.7234	0.0618	0.7480	1.4621	2.0937
ln*smpr*	Substitute meat price risk	3630	0.0000	0.9960	−2.3617	−1.8348	−0.6637	0.0067	0.7602	1.5738	2.0072
*hrzdo*	High–risk zoonotic disease outbreak	3630	0.0752	0.2638	0.0000	0.0000	0.0000	0.0000	0.0000	1.0000	1.0000
*lrzdo*	Low–risk zoonotic disease outbreak	3630	0.0146	0.1200	0.0000	0.0000	0.0000	0.0000	0.0000	0.0000	1.0000
ln*phs*	Public health scare over zoonotic disease	3630	0.0000	0.9960	−3.2898	−0.9779	−0.3994	−0.0225	0.3263	1.8017	3.0464
ln*pfp*	Poultry feed price	3630	0.0000	0.9960	−2.1926	−1.8369	−0.6490	0.1504	0.7813	1.3777	1.7045
ln*pcp*	Poultry chick price	3630	0.0000	0.9960	−2.2175	−1.6601	−0.7185	0.0805	0.7172	1.5611	2.1272
ln*apo*	Aggregate poultry output	3630	0.0000	0.9960	−2.1705	−1.7574	−0.8146	0.2220	0.8382	1.2924	1.5810
ln*upc*	Urban poultry consumption	3630	0.0000	0.9960	−3.4069	−1.5429	−0.8544	0.2819	0.7217	1.2229	1.4482
ln*rpc*	Rural poultry consumption	3630	0.0000	0.9960	−1.8106	−1.2963	−0.8385	−0.0496	0.8958	1.5392	1.6381

Notes: Summary statistics for processed data. Source: Authors’ original calculations using Stata.

**Table 3:** Panel-data unit-root tests.

Variable	Measurement	LLC	*p*-Value	IPS	*p*-Value	Fisher-ADF	*p*-Value
ln*hrmpr*	High–risk meat price risk	−1.6965 **	0.0449	−3.4318 ***	0.0003	25.1339 ***	0.0000
ln*lrmpr*	Low–risk meat price risk	−12.4630 ***	0.0000	−13.7993 ***	0.0000	35.8648 ***	0.0000
ln*smpr*	Substitute meat price risk	−19.0776 ***	0.0000	−14.8455 ***	0.0000	61.2899 ***	0.0000
ln*phs*	Public health scare over zoonotic disease	−32.0314 ***	0.0000	−37.3874 ***	0.0000	122.6781 ***	0.0000
ln*pfp*	Poultry feed price	−6.6497 ***	0.0000	−10.7574 ***	0.0000	21.4509 ***	0.0000
ln*pcp*	Poultry chick price	−14.4260 ***	0.0000	−15.6815 ***	0.0000	39.2815 ***	0.0000
ln*apo*	Aggregate poultry output	−7.7502 ***	0.0000	−8.3519 ***	0.0000	25.9374 ***	0.0000
ln*upc*	Urban poultry consumption	−14.4567 ***	0.0000	−15.2734 ***	0.0000	36.1842 ***	0.0000
ln*rpc*	Rural poultry consumption	−3.7593 ***	0.0001	−5.3501 ***	0.0000	22.6908 ***	0.0000

Notes: Panel-data unit-root tests. The table shows that all continuous variables have passed panel-data unit-root tests, that is, LLC, IPS, and Fisher-ADF tests. ** *p* < 0.05, *** *p* < 0.01. Source: Authors’ original calculations using Stata.

**Table 4:** Moran test for spatial correlation in meat price risks.

Month	H_MI	L_MI	S_MI	Month	H_MI	L_MI	S_MI
2007m11	0.1412 **	−0.1480	0.1246 **	2013m5	0.1763 **	0.0209	0.0348
2008m5	0.2494 ***	0.2556 ***	−0.1197	2013m11	0.0122	0.0430	0.0355
2008m11	−0.0682	0.1897 **	0.3710 ***	2014m5	−0.2102 **	−0.0654	0.0871 *
2009m5	0.1786 **	0.0829	0.1289 **	2014m11	−0.0961	−0.0579	−0.1457
2009m11	−0.0653	0.0047	0.0468	2015m5	0.0087	0.0194	−0.0476
2010m5	0.1073 *	0.0885*	−0.0745	2015m11	0.2862 ***	0.4130 ***	−0.1125
2010m11	0.0393	−0.0697	−0.0892	2016m5	0.3618 ***	0.2766 ***	0.1702 **
2011m5	0.0530	−0.0201	0.0797	2016m11	0.3705 ***	0.3873 ***	−0.1005
2011m11	−0.0928	0.1442 **	0.2614 ***	2017m5	−0.0265	0.1775**	−0.0269
2012m5	0.0358	0.4379 ***	−0.0231	2017m11	−0.1157	0.2356 ***	0.0735
2012m11	−0.1089	0.1106 *	−0.0009				

Notes: Moran test for spatial correlation in meat price risks (high-risk, low-risk, and substitute meat), using squared idistance matrix (*W*(1)). The table shows that meat price risks are mostly positively spatially correlated across provinces, for high-risk, low-risk, and substitute meat. Results are reported semiannually. * *p* < 0.10, ** *p* < 0.05, *** *p* < 0.01. Source: Authors’ original calculations using Stata.

**Table 5:** Benchmark analysis on Hypotheses H1–H2.

	(1)	(2)	(3)	(4)	(5)	(6)
	H_dynSDM	H_dynSAR	L_dynSDM	L_dynSAR	S_dynSDM	S_dynSAR
Panel A						
*hrzdo* (*β*_1_)	−0.0951 **	−0.1044 **	−0.0228	−0.0334	0.0152	0.0073
*lrzdo* (*β*_2_)	−0.1110 *	−0.1088 *	0.0093	0.0108	−0.0132	−0.0157
ln*phs* (*β*_3_)	−0.0177 *	−0.0266 ***	−0.0236 ***	−0.0337 ***	−0.0061 *	−0.0058 *
ln*pfp* (*β*_4_)	0.0279 **	0.0278 **	0.0179 **	0.0178**	0.0006	0.0009
ln*pcp* (*β*_5_)	0.0236 *	0.0246 **	0.0645 ***	0.0655 ***	0.0084 ***	0.0088 ***
ln*hrmpr* (*β*_6_)			0.0978 ***	0.0972 ***	0.0027	0.0025
ln*lrmpr* (*β*_7_)	0.0913 ***	0.0917 ***			0.0061	0.0060
ln*smpr* (*β*_8_)	0.0039	0.0039	0.0114	0.0114		
ln*apo* (*β*_9_)	0.0054	0.0054	−0.0287 *	−0.0288 *	0.0007	0.0008
ln*upc* (*β*_10_)	−0.0320 ***	−0.0314 ***	0.0232 *	0.0236 *	−0.0033	−0.0030
ln*rpc* (*β*_11_)	−0.0123	−0.0131	0.0183	0.0171	0.0041	0.0032
Panel B						
L.ln*hrmpr* (*τ*_1_)	0.9219 ***	0.9206 ***				
L.Wln*hrmpr* (*ψ*_1_)	−0.5431 ***	−0.5508 ***				
L.ln*lrmpr* (*τ*_2_)			0.8121 ***	0.8113 ***		
L.Wln*lrmpr* (*ψ*_2_)			−0.4748 ***	−0.4828 ***		
L.ln*smpr* (*τ*_3_)					0.8843 ***	0.8847 ***
L.Wln*smpr* (*ψ*_3_)					−0.7713 ***	−0.7723 ***
W*y* (*ρ*)	0.4693 ***	0.4790 ***	0.4913 ***	0.5019 ***	0.8781 ***	0.8787 ***
Panel C						
SR_Direct_*hrzdo*	−0.1026 **	−0.1111 **	−0.0240	−0.0350	0.0094	0.0104
SR_Direct_*lrzdo*	−0.1220 *	−0.1158 *	−0.0069	0.0114	−0.0222	−0.0226
SR_Direct_ln*phs*	−0.0205 **	−0.0270 ***	−0.0279 ***	−0.0349 ***	−0.0071 *	−0.0076 *
SR_Indirect_*hrzdo*	−0.1217	−0.0974 *	−0.0323	−0.0314	−0.1168	0.0557
SR_Indirect_*lrzdo*	−0.1672	−0.0986 *	−0.2843	0.0116	−0.1593	−0.1191
SR_Indirect_ln*phs*	−0.0706 ***	−0.0227 ***	−0.0868 ***	−0.0321 ***	−0.0260	−0.0406
LR_Direct_*hrzdo*	−1.5385	−1.7073	−0.1200	−0.1776	0.2300	0.0985
LR_Direct_*lrzdo*	−2.0319	−1.8116	0.0361	0.0577	−0.0989	−0.1764
LR_Direct_ln*phs*	−0.1787	−0.4184	−0.1269 ***	−0.1768 ***	−0.0546	−0.0649
LR_Indirect_*hrzdo*	0.4889	0.7307	−0.0540	−0.0237	3.4883	0.5625
LR_Indirect_*lrzdo*	0.7158	0.8705	−0.9297	0.0230	1.0717	−0.1847
LR_Indirect_ln*phs*	−0.2137	0.2098	−0.2346*	−0.0316	0.1712	−0.2070
Panel D						
province_FE	Yes	Yes	Yes	Yes	Yes	Yes
month_trend	Yes	Yes	Yes	Yes	Yes	Yes
cluster_robust	Yes	Yes	Yes	Yes	Yes	Yes
Observations	3600	3600	3600	3600	3600	3600
BIC	3138.4065	3119.6369	3260.1037	3245.2175	−2541.9425	−2564.2596

Notes: Benchmark analysis of local and spatial spillovers of zoonotic disease shocks to meat price risks (Hypotheses H1–H2) using QML estimator, with squared idistance matrix (*W*(1)). The table shows that (i) zoonotic disease outbreaks themselves only cause local and neighboring meat price risks for high-risk meat, not for low-risk or substitute meat; (ii) public health scares exacerbate local and neighboring meat price risks for high-risk and low-risk meat, and local meat price risks for substitute meat; and (iii) public health scares maintain long-run impacts on local meat price risks for low-risk meat. Panel A reports main coefficients (*β*). Panel B reports temporal coefficients (*τ*), spatiotemporal coefficients (*ψ*), and spatial coefficients (*ρ*). Panel C reports short-run local spillovers (SR_Direct), short-run spatial spillovers (SR_Indirect), long-run local spillovers (LR_Direct), and long-run spatial spillovers (LR_Indirect). Panel D reports model statistics. * *p* < 0.10, ** *p* < 0.05, *** *p* < 0.01. Source: Authors’ original calculations using Stata.

**Table 6:** Further analysis on Hypotheses H3–H4.

Distance	Spatial Spillover Measures (High-Risk Meat)	*p*-Value	Spatial Spillover Measures (Low-Risk Meat)	*p*-Value	Distance	Spatial Spillover Measures (High-Risk Meat)	*p*-Value	Spatial Spillover Measures (Low-Risk Meat)	*p*-Value
100	−0.0243	0.0025	−0.0337	0.0000	1600	−0.0264	0.0001	−0.0281	0.0000
200	−0.0266	0.0069	−0.0360	0.0001	1700	−0.0302	0.0007	−0.0287	0.0000
300	−0.0274	0.0119	−0.0381	0.0002	1800	−0.0186	0.0001	−0.0247	0.0000
400	−0.0276	0.0179	−0.0394	0.0003	1900	−0.0182	0.0000	−0.0224	0.0000
500	−0.0281	0.0101	−0.0403	0.0004	2000	−0.0166	0.0000	−0.0182	0.0000
600	−0.0274	0.0069	−0.0391	0.0003	2100	−0.0126	0.0000	−0.0396	0.0000
700	−0.0273	0.0037	−0.0399	0.0002	2200	−0.0112	0.0000	−0.0165	0.0000
800	−0.0276	0.0020	−0.0403	0.0001	2300	−0.0097	0.0000	−0.0139	0.0000
900	−0.0263	0.0009	−0.0397	0.0001	2400	−0.0087	0.0000	−0.0130	0.0000
1000	−0.0260	0.0010	−0.0363	0.0002	2500	−0.0091	0.0000	−0.0117	0.0000
1100	−0.0253	0.0010	−0.0342	0.0001	2600	−0.0077	0.0000	−0.0103	0.0000
1200	−0.0283	0.0005	−0.0336	0.0000	2700	−0.0078	0.0000	−0.0092	0.0000
1300	−0.0281	0.0005	−0.0306	0.0000	2800	−0.0059	0.3792	−0.0129	0.0000
1400	−0.0221	0.0002	−0.0294	0.0000	2900	−0.0074	0.0000	−0.0078	0.0000
1500	−0.0242	0.0001	−0.0304	0.0000	3000	−0.0059	0.0000	−0.0078	0.0000

Notes: Further analysis of distance-decaying spillovers of public health scares to meat price risks (Hypotheses H3–H4), using distance-varying spatial weighting matrix (squared idistance), $W_{d}^{v(s)}$. Table reports distance effects on spatial spillover measures, which are heterogeneous in high- and low-risk meat. We run regressions of high- and low-risk meat price risks 147 times, respectively, from 80 km to 3000 km with 20 km as incremental value, implementing dynamic SAR. Spatial spillover measures are calculated for every 20 km threshold (147 observations), but only reported for every 100 km threshold for space considerations (30 observations). We winsorize all spatial spillover measures at the 3% level to eliminate outliers. Spatial spillover measures = short-run spatial spillover effects of public health scares on meat price risks, at different distance thresholds. Source: Authors’ original calculations using Stata.

**Table 7:** Further analysis on Hypotheses H3–H4 (robustness).

Distance	Spatial Spillover Measures (High-Risk Meat)	*p*-Value	Spatial Spillover Measures (Low-Risk Meat)	*p*-Value	Distance	Spatial Spillover Measures (High-Risk Meat)	*p*-Value	Spatial Spillover Measures (Low-Risk Meat)	*p*-Value
100	−0.0142	0.0000	−0.0189	0.0000	1600	−0.0135	0.0000	−0.0130	0.0000
200	−0.0143	0.0001	−0.0173	0.0000	1700	−0.0109	0.0000	−0.0130	0.0000
300	−0.0145	0.0004	−0.0181	0.0000	1800	−0.0105	0.0001	−0.0144	0.0000
400	−0.0158	0.0006	−0.0187	0.0000	1900	−0.0108	0.0000	−0.0165	0.0000
500	−0.0168	0.0004	−0.0206	0.0000	2000	−0.0098	0.0000	−0.0108	0.0000
600	−0.0176	0.0004	−0.0213	0.0000	2100	−0.0096	0.0000	−0.0122	0.0000
700	−0.0144	0.0006	−0.0203	0.0000	2200	−0.0089	0.0000	−0.0248	0.0000
800	−0.0161	0.0001	−0.0222	0.0000	2300	−0.0066	0.0000	−0.0096	0.0000
900	−0.0158	0.0000	−0.0248	0.0000	2400	−0.0063	0.0000	−0.0087	0.0000
1000	−0.0139	0.0001	−0.0228	0.0000	2500	−0.0051	0.0000	−0.0077	0.0000
1100	−0.0115	0.0003	−0.0192	0.0000	2600	−0.0065	0.0000	−0.0073	0.0000
1200	−0.0143	0.0000	−0.0183	0.0000	2700	−0.0306	0.0000	−0.0087	0.0000
1300	−0.0140	0.0000	−0.0154	0.0000	2800	−0.0063	0.0000	−0.0080	0.0000
1400	−0.0089	0.0000	−0.0140	0.0000	2900	−0.0059	0.0000	−0.0078	0.0000
1500	−0.0118	0.0000	−0.0171	0.0000	3000	−0.0051	0.0000	−0.0073	0.0000

Notes: Further analysis of distance-decaying spillovers of public health scares to meat price risks (Hypotheses H3–H4) (robustness), using distance-varying spatial weighting matrix (exponential idistance), $W_{d}^{v(e)}$. Table reports distance effects on spatial spillover measures, which are heterogeneous in high- and low-risk meat. We run regressions of high- and low-risk meat price risks 147 times, respectively, from 80 km to 3000 km with 20 km as incremental value, implementing dynamic SAR. Spatial spillover measures are calculated for every 20 km threshold (147 observations), but only reported for every 100 km threshold for space considerations (30 observations). We winsorize all spatial spillover measures at the 3% level to eliminate outliers. Spatial spillover measures = short-run spatial spillover effects of public health scares on meat price risks, at different distance thresholds. Source: Authors’ original calculations using Stata.
